# Systematic analysis of anoikis-related genes identifies SRPX2-FAK/AKT-IL-6 axis in the progression and peritoneal metastasis of gastric cancer

**DOI:** 10.3389/fgene.2025.1736097

**Published:** 2026-01-08

**Authors:** Dong Hou, Jinhao Yu, Yequan Xie, Shengning Zhou, Jintao Zeng, Mingtao Liang, Fang Zheng, Jianan Tan, Fanghai Han

**Affiliations:** 1 Department of Gastrointestinal Surgery, Sun Yat-sen Memorial Hospital, Sun Yat-sen University, Guangzhou, Guangdong, China; 2 Department of Gastrointestinal Surgery, The Affiliated Guangdong Second Provincial General Hospital of Jinan University, Guangzhou, Guangdong, China; 3 Department of Medical Research Center, Sun Yat-sen Memorial Hospital, Sun Yat-sen University, Guangzhou, Guangdong, China; 4 Department of Guangdong Provincial Key Laboratory of Malignant Tumor Epigenetics and Gene Regulation, Medical Research Center, Sun Yat-sen Memorial Hospital, Sun Yat-sen University, Guangzhou, Guangdong, China

**Keywords:** anoikis, cancer-associated fibroblasts (CAF), gastric cancer, peritoneal metastasis, SRPX2

## Abstract

**Introduction:**

Peritoneal metastasis (PM) remains a primary cause of poor prognosis in advanced gastric cancer (GC). While anoikis resistance enables detached tumor cells to survive and promotes invasion and metastasis, its specific mechanisms in GC related PM are not yet fully understood.

**Methods:**

Anoikis related differentially expressed genes were identified using GC samples from the TCGA and GEO databases. Molecular subtypes were characterized by non negative matrix factorization (NMF) clustering. Survival outcomes were assessed via Kaplan–Meier analysis, and functional profiles were evaluated through pathway enrichment scoring. A prognostic risk model was constructed by combining weighted gene co expression network analysis (WGCNA) with Lasso–Cox regression. For PM diagnosis, characteristic genes were screened using support vector machine (SVM) and random forest (RF) algorithms to build a diagnostic model. Experimental validation was performed to confirm the expression and functional role of SRPX2.

**Results:**

GC was stratified into two molecular subtypes. Subtype A showed marked enrichment of anoikis resistance related factors and was associated with significantly poorer prognosis. A six gene prognostic signature (HEYL, SRPX2, LBH, PLAT, ITGAV, HTRA1) was established and validated externally. A nine gene diagnostic panel (SLC30A9, ZFHX4, CYTB, NDFIP2, NMNAT2, SRPX2, TBC1D8B, CLEC3B, CHRDL2) was also constructed for PM. SRPX2 was identified as an independent prognostic marker and a PM associated biomarker, highly expressed in cancer associated fibroblasts (CAFs). Functional studies demonstrated that SRPX2 promotes GC progression and peritoneal metastasis by activating the FAK/AKT pathway and IL-6 paracrine signaling, thereby inducing anoikis resistance.

**Discussion:**

This study proposes an anoikis based molecular classification of GC and develops both prognostic and diagnostic models for PM. It further reveals that SRPX2 driven anoikis resistance, mediated through the FAK/AKT-IL-6 axis, facilitates peritoneal metastasis. These findings provide new directions for prognostic assessment and therapeutic strategies in gastric cancer.

## Introduction

Gastric cancer (GC) is a common malignant tumor that poses a serious threat to human health. In 2022, there were over 968,000 new cases of GC worldwide and nearly 660,000 deaths, ranking it fifth in both incidence and cancer-related mortality (Sung et al., 2021). The incidence of GC exhibits regional variation, with the highest rates observed in East Asia and Eastern Europe (Yeoh and Tan, 2022). In recent years, due to *Helicobacter pylori* eradication, early cancer screening, and optimized diagnosis and treatment strategies, the overall incidence and mortality rates of GC have shown a gradual decline (Smyth et al., 2020). However, due to its insidious early symptoms, the majority of patients are diagnosed at an advanced stage with distant metastasis, resulting in a poor prognosis (Sexton et al., 2020). The 5-year survival rate for metastatic GC patients is less than 10% (Chen et al., 2016). The peritoneum is a common site of metastasis, with approximately 53%–66% of advanced GC patients developing peritoneal metastasis (PM), which poses a significant challenge to conventional treatment strategies (Dong et al., 2019). With advances in imaging technology, numerous studies have developed individualized prediction models for PM by screening effective radiomic features (Dong et al., 2019). Nevertheless, research on the molecular mechanisms underlying peritoneal implantation and metastasis in GC remains limited. Therefore, identifying biomarkers that can effectively predict poor prognosis and the risk of PM is of great significance for selecting therapeutic targets and guiding clinical decision-making in advanced GC.

When cells detach from the extracellular matrix (ECM) due to lack of or aberrant adhesion, they are eliminated through a specific form of apoptosis termed “anoikis” (derived from the Greek word for “homelessness”) (Liotta and Kohn, 2004). This process maintains tissue homeostasis by removing misplaced cells. The progression of tumor metastasis typically involves steps such as stromal separation, local invasion, migration, survival in circulation, extravasation, and colonization at secondary sites (Lu et al., 2015). The acquisition of anoikis resistance by malignant cells is a prerequisite for metastasis (Kim et al., 2012; Simpson et al., 2008). Consequently, growing attention has been directed toward the role of anoikis resistance in tumorigenesis and progression. For instance, collagen IV/integrin interactions in the stroma activate anoikis resistance via B-cell lymphoma (BCL) family proteins, providing important clues for research on liver metastasis (Burnier et al., 2011). Upregulation of glutamate dehydrogenase 1 (GDH1) mediated by the transcription factor PLAG1 promotes lung cancer metastasis by inducing anoikis resistance through the CamKK2–AMPK signaling pathway (Jin et al., 2018). Clinically prognostic models based on anoikis-related genes have been validated in multiple cancers (Chi et al., 2022; He et al., 2023). However, the predictive value of these genes for GC prognosis and peritoneal metastasis has not been systematically investigated. At the same time, the promotive role of the tumor stroma in malignant progression has recently garnered increasing attention. Various stromal cells in the tumor microenvironment are widely recognized to directly influence the malignant characteristics of adjacent tumor cells (Hanahan and Weinberg, 2011). In particular, cancer-associated fibroblasts (CAFs), as major components of the tumor stroma (Yamaguchi and Sakai, 2015), interact with tumor cells and often induce aggressive phenotypes, promoting metastatic potential and chemotherapy resistance (Duluc et al., 2015). However, less is known about another aspect of CAF-mediated regulation: the activation of apoptosis resistance, which warrants further exploration.

SRPX2 (Sushi-repeat-containing protein X-linked 2) is a chondroitin sulfate proteoglycan initially identified as a downstream target of E2A–PBX1 in leukemia (Kurosawa et al., 1999). It is involved in brain development, language processing, and angiogenesis (Reinthaler et al., 2014; Liu et al., 2017). In tumor tissues and cells, SRPX2 expression is upregulated through multiple signaling pathways and influences tumor cell proliferation, migration, invasion, and chemosensitivity (Wang et al., 2021; Liu et al., 2015). Recent studies have identified molecular pathways regulating anoikis resistance, including cell adhesion molecules and growth factors that promote epithelial–mesenchymal transition (Adeshakin et al., 2021). Key downstream pathways such as focal adhesion and PI3K/Akt play important roles in anti-apoptosis and tumor proliferation (Moro et al., 2009). Although studies suggest that SRPX2 can increase FAK phosphorylation (Tanaka et al., 2009), its role in activating CAFs within the GC tumor microenvironment and its specific mechanism in regulating anoikis resistance in tumor cells require further investigation.

In this study, we performed unsupervised clustering of anoikis-related differentially expressed genes in GC cohorts from TCGA and GEO databases using non-negative matrix factorization. A prognostic model for GC was constructed using weighted gene co-expression network analysis (WGCNA). Key genes associated with peritoneal metastasis were screened using machine learning algorithms such as SVM and RF, and a predictive model was established. Ultimately, SRPX2 was identified as an important biomarker for GC prognosis and peritoneal metastasis, offering a potential target for therapeutic intervention and suppression of PM progression in GC.

## Materials and methods

Please find the complete Methods and Materials in Additional file 1.

### Data acquisition and processing

Transcriptomic data and corresponding clinical information of gastric cancer (GC) patients were obtained from The Cancer Genome Atlas (TCGA) database (https://portal.gdc.cancer.gov/). Samples with survival time ≤0 days or missing survival information were excluded. To expand the dataset, expression profiles from 32 paired GC and normal tissues in the Gene Expression Omnibus (GEO) dataset GSE65801 (www.ncbi.nlm.nih.gov/geo/) were used for differential gene screening. Additionally, datasets GSE84426, GSE84433, and GSE38749 were included for constructing and validating prognostic risk models. To investigate factors associated with peritoneal metastasis (PM) in GC, sequencing data from the GSE15081 dataset were obtained, which included 75 patients without PM and 33 patients with PM for subsequent analysis. Single-cell sequencing data of peritoneal metastasis samples from GSE163558 and GSE183904 were integrated for further analysis. Using GeneCards (https://www.genecards.org/), 506 potential genes related to anoikis resistance were identified with a relevance score >0.4. Differential expression analysis was performed using the “DESeq2” package with thresholds set at |log_2_FC| > 1 and FDR <0.05.

### Cell culture

The GC cell lines AGS, HGC-27, and MKN45 were purchased from the American Type Culture Collection (ATCC, Manassas, VA, United States) and maintained in the Gastrointestinal Oncology Laboratory at Sun Yat-sen Memorial Hospital, Sun Yat-sen University. Normal fibroblasts (NFs) and cancer-associated fibroblasts (CAFs) were isolated from fresh human normal adjacent tissues and primary tumor tissues, respectively. GC cell lines were cultured in DMEM medium (Gibco, Carlsbad, CA, United States) supplemented with 10% fetal bovine serum (Invitrogen, Carlsbad, CA, United States). Isolated CAFs and NFs were cultured in fibroblast-specific medium (ScienCell Research Laboratories, Carlsbad, CA, United States) supplemented with 2.5% FBS and 1% growth factors. All cells were maintained at 37 °C in a humidified atmosphere with 5% CO_2_. Experiments were conducted during the logarithmic growth phase of the cells.

### Patient specimens and informed consent

From June 2023 to June 2024, primary tumor tissues and matched adjacent normal tissues were collected from six patients who underwent gastric cancer resection at Sun Yat-sen Memorial Hospital (Guangzhou, China). All patients were clinically diagnosed with gastric cancer (GC) and confirmed by postoperative pathological examination. Among them, three cases were diagnosed with synchronous peritoneal metastasis, while the other three had no peritoneal or distant metastasis. Residual specimens remaining after routine pathological examination were preserved in liquid nitrogen for subsequent research use. This study was approved by the Ethics Committee of Sun Yat-sen Memorial Hospital (SYSKY-2024-887-01) and conducted in accordance with the principles of the Declaration of Helsinki. Written informed consent was obtained from all patients, authorizing the use of donated samples and associated information for all medical research purposes.

### Animal model establishment

All animal care and experimental procedures were reviewed and approved by the Sun Yat-sen University Institutional Animal Care and Use Committee (SYSU-IACUC-2024-002900), in compliance with animal protection principles, welfare guidelines, and ethical standards as well as national regulations on laboratory animal welfare. Five-week-old male BALB/c nude mice were purchased from GemPharmatech Co., Ltd. (Nanjing, China). In the xenograft tumor model, cells were suspended in 50 μL of a Matrigel/PBS mixture. A total of 1 × 10^6^ MKN-45 cells mixed with 1 × 10^6^ fibroblasts (Vector group, n = 5; shSRPX2 group, n = 5) were subcutaneously injected into the flanks of the mice. Tumor volume and body weight were monitored throughout the study period using digital calipers. Tumor volume was calculated using the formula: Volume (mm^3^) = (length × width (Yeoh and Tan, 2022))/2. After 4 weeks, the mice were anesthetized via inhalation of isoflurane (oxygen flow rate: 0.3–0.5 L/min, isoflurane concentration: 3%–4%) and euthanized by cervical dislocation. Resected tumor tissues were stored at −80 °C for subsequent immunohistochemical (IHC) analysis. To inhibit IL-6 signaling, a xenograft model was established by subcutaneously injecting a mixture of 1 × 10^6^ MKN-45 cells and 1 × 10^6^ fibroblasts (Vector group or OE-SRPX2 group) suspended in 50 μL of Matrigel/PBS. In the OE-SRPX2 CAF co-injection group, mice received intraperitoneal injections of either tocilizumab (10 μg/g, Actemra, Genentech, n = 5) or IgG control (10 μg/g, Sigma, n = 5). Tumor volume and body weight were monitored regularly. The mice were anesthetized and euthanized by cervical dislocation using the same method described above, and tumor tissues were collected for further analysis. In the peritoneal metastasis model, luciferase-EGFP-expressing MKN-45 cells along with treated CAFs were suspended in 50 μL of Matrigel/PBS and injected intraperitoneally. Metastasis was monitored using an IVIS imaging system. At the end of the experiment, mice were anesthetized and euthanized by cervical dislocation. Abdominal metastatic nodules were dissected and collected for subsequent analysis.

### Statistical analysis

Statistical analyses were performed using GraphPad Prism 9.0. Statistical results are presented as mean ± S.E.M. or mean ± SD from at least three independent experiments. Group differences were compared using Student’s two-tailed t-test and one-way ANOVA. Spearman correlation analysis was used to assess intergroup correlations. *P* < 0.05 was considered statistically significant. *, *P* < 0.05; **, *P* < 0.01; ***, *P* < 0.001.

## Results

### Identification of anoikis-related differential genes and genetic variation landscape in GC

We screened 506 anoikis-related genes (ARGs) from the GeneCards database using a relevance score >0.4 as the selection criterion. Subsequently, using the “DESeq2” package with |log FC| > 1 and FDR <0.05 as cut-off criteria, we identified 87 ARGs with significant differential expression between tumor and normal samples from the TCGA-STAD cohort (Supplementary Figures S1A,B). To further ensure screening accuracy, we obtained 2,265 differential genes from sequencing data of 32 paired gastric cancer and normal tissues in the GSE65801 dataset based on the same criteria (Supplementary Figures S1C). Among them, 42 overlapping ARGs were identified Supplementary Figures S1D,E). Somatic mutation analysis revealed that 134 (31.09%) samples in the TCGA-STAD cohort underwent genetic alterations in anoikis-related factors, mainly including missense mutations and frameshift deletions (Supplementary Figure S1F). Additionally, copy number variation (CNV) analysis showed that CNV was prevalent in differentially expressed ARGs in gastric cancer samples, with CLDN1, ETV4, and LAMC2 mainly showing amplification variations, while EZH2, EPHB6, and SERPINB5 exhibited significant deletion frequencies (Supplementary Figure S1G). The circle plot displays the specific chromosomal locations of mutations in ARGs (Supplementary Figure S1H). Therefore, anoikis-related genes have the potential to serve as diagnostic biomarkers for GC.

### Identification of anoikis-related subclusters in GC

To expand the sample size for prognostic studies, we standardized and merged the TCGA-STAD cohort and GSE84433 dataset into an ARG research cohort for subsequent studies. Univariate COX analysis showed that most of the differentially expressed ARGs were significant risk factors and correlated with each other (Supplementary Figure S2A). Among them, 14 factors had significant prognostic value, including CLDN1, MMP11, EZH2, TIMP1, PBK, LAMC2, SERPINE1, THY1, TNFRSF12A, NOTCH3, ANGPTL4, PDGFRB, CDX2, and CCDC80 (Supplementary Figure S2B). To further identify the potential mechanisms of anoikis in GC, we used the Non-negative Matrix Factorization (NMF) algorithm to identify characteristic subclusters in GC patient samples from research cohort A based on the 14 prognosis-related ARGs. The “brunet” algorithm was used by default to select the optimal number of clusters based on cophenetic correlation (Supplementary Figure S2C,D). Research cohort A was divided into clusters A and B, and principal component analysis (PCA), Uniform Manifold Approximation and Projection (UMAP), and t-distributed Stochastic Neighbor Embedding (t-SNE) plots demonstrated that the characteristic subpopulations were well-differentiated and clustered (Supplementary Figure S2E). The Kaplan-Meier survival curve showed that the prognosis of cluster A was significantly better than that of cluster B (*P* = 0.002; Supplementary Figure S2F). Subsequently, we conducted a differential analysis of the 14 prognosis-related ARGs between different subtypes. As shown in the figure, anoikis resistance-related genes such as MMP11, TIMP1, SERPINE1, THY1, TNFRSF12A, NOTCH3, ANGPTL4, PDGFRB, and CCDC80 were significantly upregulated in cluster A (Supplementary Figure S2G). The heatmap displays the expression and clinical characteristics of prognosis-related ARGs in different subclusters (Supplementary Figure S2H).

### Immune characteristics and functional analysis between anoikis characteristic subclusters

Furthermore, we compared the immune phenotypic characteristics of different subclusters. The infiltration abundance of activated B cells, activated CD8^+^ T cells, macrophages, and NK cells was significantly higher in cluster A than in cluster B (Supplementary Figure S2I). Therefore, the poor prognosis of subtype A may be related to the immune-inflammatory microenvironment. To further explore the potential biological dysfunction of different subtypes, we performed Gene Set Variation Analysis (GSVA) and Gene Set Enrichment Analysis (GSEA) on the two subtypes. GSVA showed that pathways such as cell adhesion molecules (CAMs), focal adhesion, and ECM receptor interaction were significantly enriched in cluster A compared to cluster B (Supplementary Figure S2J). The enriched pathways in GSEA analysis again validated the enrichment of related pathways in cluster A (Supplementary Figure S2K). Therefore, the enrichment of these pathways may be related to poor prognosis and the activation mechanism of anoikis resistance. This provides direction for our subsequent downstream mechanism exploration.

### Construction of weighted gene Co-expression network and identification of anoikis characteristic modules

To more accurately identify anoikis-related signature genes associated with gastric cancer progression, we first performed differential expression analysis between different subtypes, yielding a total of 1,344 differentially expressed genes (DEGs). Subsequently, a weighted gene co-expression network was constructed based on these DEGs using the expression profiles from the ARG study cohort. After sample clustering to exclude outlier samples (Supplementary Figure S3A), a soft threshold power of β = 11 was selected (Supplementary Figure S3B). A topological overlap matrix was then transformed, and a dendrogram was generated using average linkage hierarchical clustering with a merge cut height of 0.2 and a minimum module size of 60 (Supplementary Figure S3C). This process resulted in five co-expression modules: yellow, brown, blue, turquoise, and grey, with the grey module comprising all unassigned genes. Heatmap analysis revealed that the blue module was significantly correlated with the subtype A signature (R = 0.54, p = 7e−57) (Supplementary Figure S3D). A scatter plot of module membership (MM) *versus* gene significance (GS) further confirmed a strong correlation within the blue module (cor = 0.75, p = 8.6e−32) (Supplementary Figure S3E). Therefore, 169 genes from the blue module were selected for subsequent analysis.

### Construction and validation of an anoikis-related prognostic risk model

**FIGURE 1:**
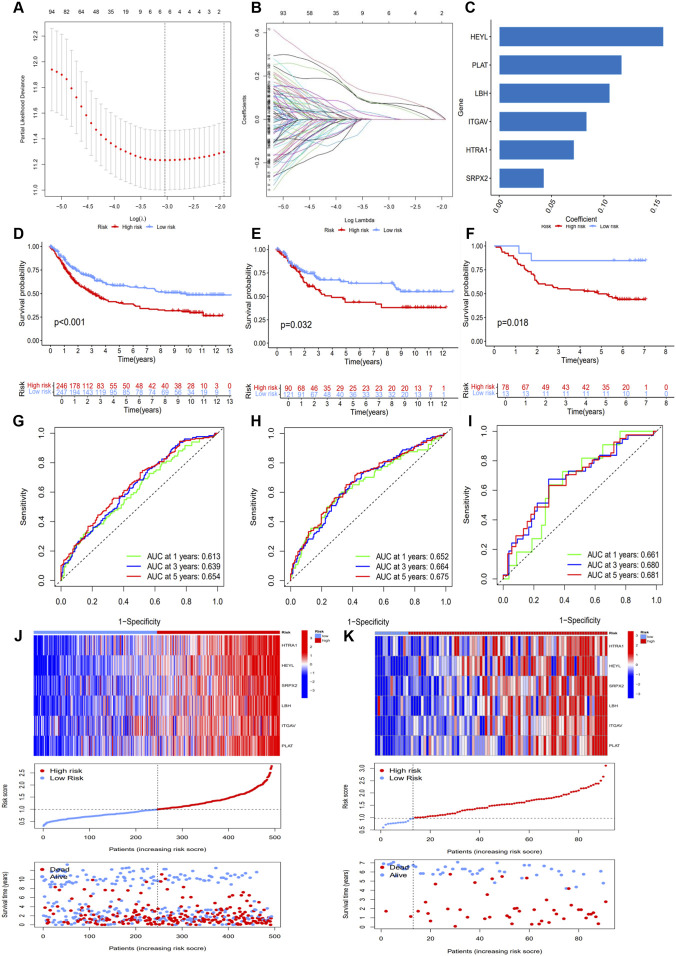
Construction of a Prognostic Risk Model Based on Anoikis-Related Module Genes. **(A)** LASSO coefficient profiles of 129 prognostic module genes. The vertical line indicates the value with minimum cross-validation error. **(B)** Trajectory of LAS variable coefficients with increasing lambda. **(C)** Risk coefficients of each gene included in the prognostic signature. **(D–F)** Kaplan–Meier survival curves between high- and low-risk groups in the internal training, internal validation, and external validation sets. **(G–I)** Time-dependent ROC curves predicting 1-, 3-, and 5-year overall survival in the internal training, internal validation, and external validation sets. **(J,K)** Expression heatmap of signature genes, distribution of risk scores, and survival status scatter plot in the internal training and external validation sets.

To elucidate the prognostic value of anoikis-related signature genes in gastric cancer (GC), we constructed a prognostic risk scoring model based on the 169 significantly correlated module genes identified in previous analyses. The ARG study cohort was first randomly divided into an internal training set (n = 493) and an internal validation set (n = 211) at a 7:3 ratio. Additionally, the GSE84426 and GSE38749 datasets were standardized and merged to serve as an external validation set (n = 91). Optimal prognostic signature factors were screened via LASSO analysis. Based on minimum partial likelihood deviation, six optimal factors—HTRA1, HEYL, SRPX2, LBH, ITGAV, and PLAT—were selected for multivariate Cox analysis to build the prognostic risk model (Figures 1A,B). As shown, all six key factors were identified as high-risk factors (Figure 1C). The risk score was calculated as follows:

$$\text{Risk Score}=\left(0.0714838946470207\times \text{HTRA}1\right)+\left(0.156841470971057\times \text{HEYL}\right)+\left(0.0427683260769306\times \text{SRPX}2\right)+\left(0.105640856086873\times \text{LBH}\right)+\left(0.0835692421728162\times \text{ITGAV}\right)+\left(0.117071070606207\times \text{PLAT}\right)$$

To evaluate model performance, samples in the internal training, internal validation, and external validation sets were divided into high- and low-risk groups according to the median risk score. Kaplan–Meier survival curves showed that the high-risk groups had significantly shorter overall survival (OS) in the internal training set (p < 0.001), internal validation set (p = 0.032), and external validation set (p = 0.018) (Figures 1D–F). The area under the curve (AUC) values for the 1-, 3-, and 5-year ROC curves in the internal training set were 0.613, 0.639, and 0.654, respectively (Figure 1G). The AUC values for the internal validation set (0.652, 0.664, 0.675) and external validation set (0.661, 0.680, 0.681) further confirmed the accuracy of the model (Figures 1H,I). Heatmaps indicated significant overexpression of model risk factors in the high-risk group of the training set (Figure 1J). Scatter plots demonstrated that high-risk patients had lower survival probabilities and earlier mortality, a trend consistently observed in the validation sets (Figure 1K, Supplementary Figure S4A). These results demonstrate that the anoikis-related risk model serves as an effective tool for prognostic prediction in GC patients.

### Correlation analysis between the prognostic model and Clinicopathological Features

**FIGURE 2:**
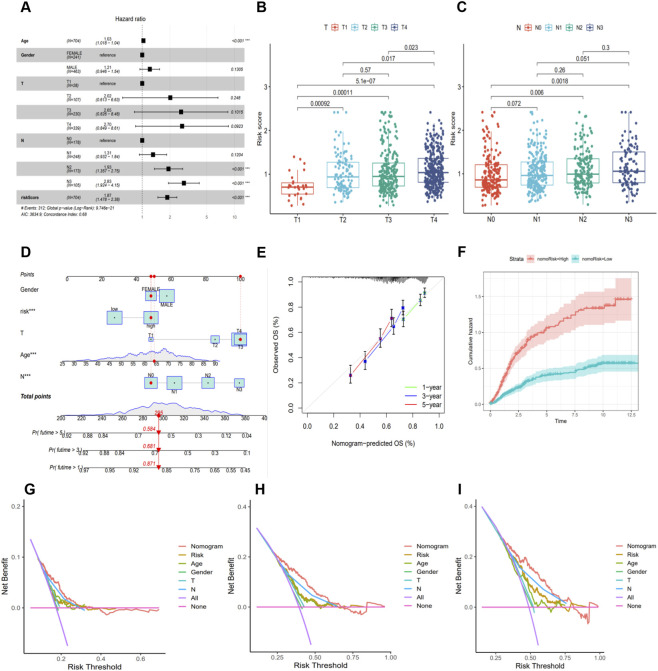
Correlation Between the Prognostic Signature and Clinicopathological Features. **(A)** Univariate Cox regression analysis of the risk score and clinical variables. **(B,C)** Box plots comparing risk scores across different T and N stages. **(D)** Nomogram integrating clinical factors and risk score to predict patient survival probability. **(E)** Calibration curve of the nomogram. **(F)** Cumulative hazard curve between high- and low-risk groups. **(G-I)** Decision curve analysis (DCA) of the nomogram for predicting 1-, 3-, and 5-year survival in the study cohort.

To investigate the relationship between the anoikis-related prognostic model and the clinicopathological characteristics of gastric cancer (GC) patients, we first compiled and screened the clinicopathological information of patients in the training and validation sets (Table.1) for subsequent analysis. Univariate and multivariate Cox regression analyses were performed to evaluate the prognostic impact of the risk score and clinical features on GC patients. The results indicated that age, N stage, and risk score were all independent prognostic factors (Figure 2A). Boxplots of risk scores stratified by different clinical features revealed significant differences in risk scores among groups with different T and N stages (Figures 2B,C; Supplementary Figure S4B,C). To provide an intuitive tool for clinical prognostic assessment, we constructed a multivariate nomogram integrating age, sex, T stage, N stage, and risk score (Figure 2D). The calibration curve demonstrated that the nomogram accurately predicted long-term survival probabilities (Figure 2E), while the cumulative hazard plot indicated a higher cumulative risk in the high-risk group (Figure 2F). Decision curve analysis (DCA) was used to evaluate the clinical utility of the nomogram. The results showed that, across 1-, 3-, and 5-year predictions, the nomogram provided higher net clinical benefits under various threshold probabilities, confirming its strong potential for clinical application (Figures 2G–I).

**TABLE 1:** Clinicopathological information of patients in intral-training set and intral-validation set.

Character	Type	Total	Intra-training set	Intra-validation set	*P* value
Age	≤65	394 (55.97%)	275 (55.78%)	119 (56.4%)	0.9456
	>65	310 (44.03%)	218 (44.22%)	92 (43.6%)	​
Gender	Female	241 (34.23%)	181 (36.71%)	60 (28.44%)	0.042*
	Male	463 (65.77%)	312 (63.29%)	151 (71.56%)	​
T	T1	28 (3.98%)	16 (3.25%)	12 (5.69%)	0.4883
	T2	107 (15.2%)	77 (15.62%)	30 (14.22%)	​
	T3	230 (32.67%)	162 (32.86%)	68 (32.23%)	​
	T4	339 (48.15%)	238 (48.28%)	101 (47.87%)	​
N	N0	178 (25.28%)	121 (24.54%)	57 (27.01%)	0.4338
	N1	248 (35.23%)	180 (36.51%)	68 (32.23%)	​
	N2	173 (24.57%)	124 (25.15%)	49 (23.22%)	​
	N3	105 (14.91%)	68 (13.79%)	37 (17.54%)	​

*Differences among variable were assessed by the chi-square test, ^*^P < 0.05.

### Development of a machine learning-based diagnostic model for peritoneal metastasis in gastric cancer

**FIGURE 3:**
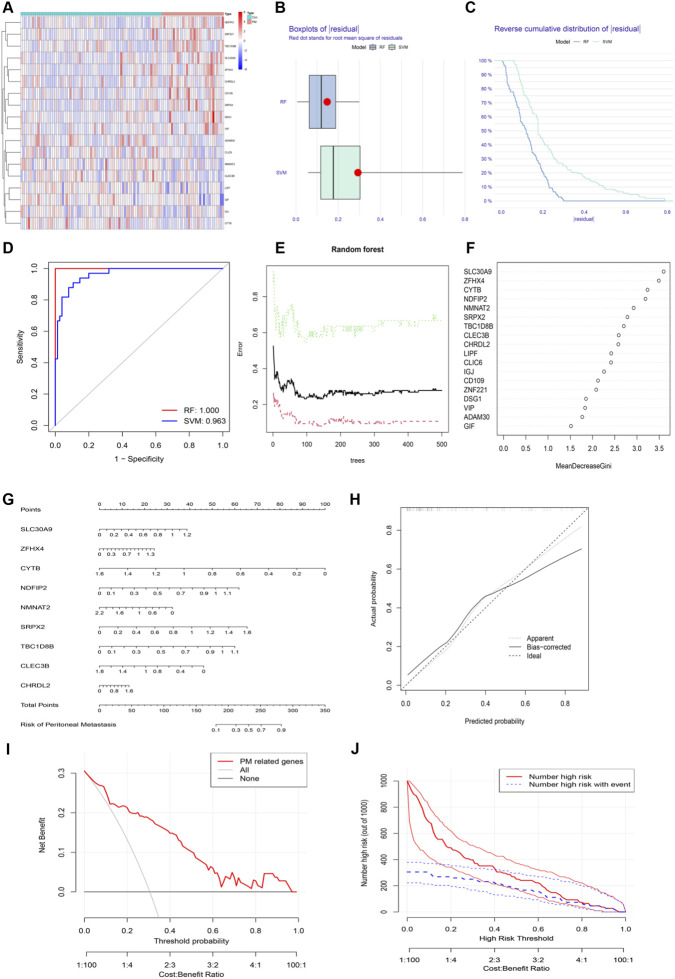
Screening of Characteristic Genes for GC Peritoneal Metastasis and Diagnostic Model Construction. **(A)** Heatmap of differentially expressed genes between peritoneal metastasis (PM) and non-PM samples in the GSE15081 dataset. **(B,C)** Residual distribution plots and cumulative error rates from feature selection using SVM and RF algorithms. **(D)** ROC curves comparing the performance of SVM and RF models. **(E,F)** RF algorithm output and gene importance ranking. **(G)** Nomogram for diagnosing GC peritoneal metastasis. The left axis indicates point assignment per gene; the bottom axis shows the total points and corresponding metastasis probability. **(H)** Calibration curve of the diagnostic model. The dashed diagonal represents ideal prediction. **(I)** Decision curve analysis (DCA) evaluating the clinical net benefit of the model. **(J)** Clinical impact curve: the red curve shows the number of patients classified as high-risk across threshold probabilities; the blue curve indicates true positives.

Previous studies have indicated that the activation of anoikis resistance in gastric cancer cells is positively correlated with peritoneal metastasis (Ye et al., 2020). To explore whether potential biomarkers of peritoneal metastasis in gastric cancer (GC) patients are associated with the activation of anoikis resistance, we analyzed sequencing data from the GSE15081 dataset, which included 33 patients with peritoneal recurrence (PR) and 75 without peritoneal recurrence (NPR). Differential expression analysis identified 18 differentially expressed genes (|log_2_FC| > 0, p < 0.05) (Figure 3A). Subsequently, both SVM-RFE and random forest (RF) algorithms were used to construct predictive models for peritoneal metastasis progression based on these differentially expressed genes. Performance comparison revealed that the model fitted with the RF algorithm achieved higher predictive accuracy (Figures 3B–D). Therefore, the RF algorithm was employed to fit clinical characteristics associated with peritoneal recurrence, and the importance of relevant genes was ranked (Figures 3E,F). Using an importance score threshold >2, nine feature genes were selected. To enhance the clinical applicability of the model, a nomogram was developed. The results showed that when the total score of the disease feature genes reached 280 points, the risk ratio for peritoneal metastasis in GC patients was as high as 90% (Figure 3G). The calibration curve demonstrated strong accuracy of the model (Figure 3H), and decision curve analysis (DCA) indicated substantial clinical net benefit across a wide range of threshold probabilities (Figure 3I). The clinical impact curve (CIC) revealed a high concordance between predicted risk and actual events when the threshold probability exceeded 0.6, suggesting excellent clinical predictive performance of the model (Figure 3J). Notably, SRPX2 was consistently identified both as a risk factor in the prognostic model and as a feature gene in the peritoneal metastasis model. We therefore hypothesize that high expression of SRPX2 may be associated with the activation of anoikis resistance in GC cells, thereby contributing to peritoneal metastasis and poor prognosis.

### SRPX2 is highly expressed in GC tissues and correlates with CAF infiltration in the TME

**FIGURE 4:**
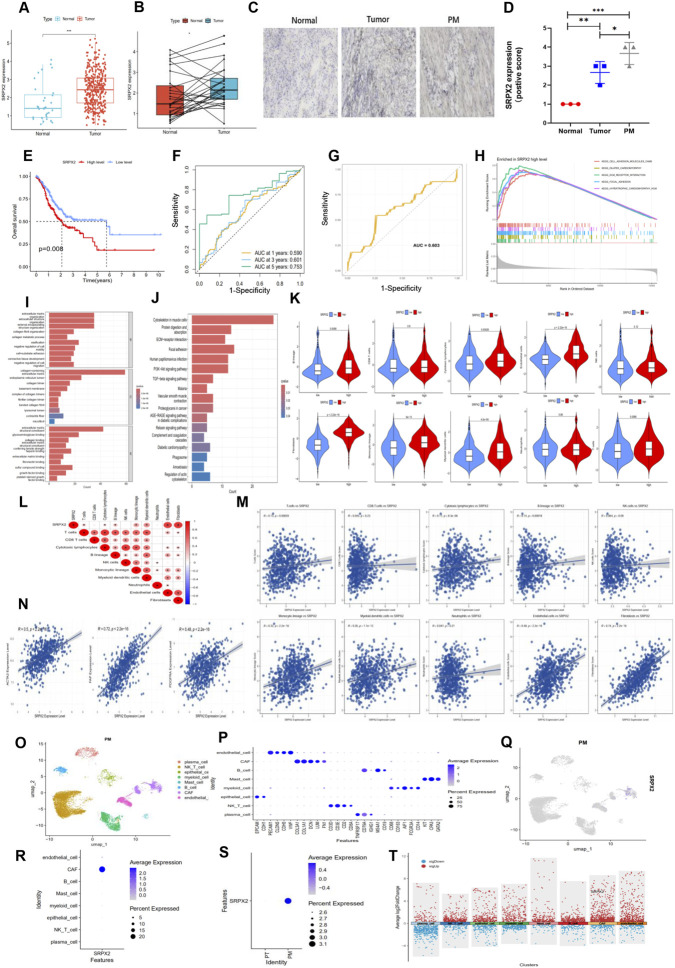
SRPX2 is highly expressed in GC tissues and correlates with CAF infiltration. **(A)** SRPX2 mRNA expression is elevated in tumor tissues compared to normal tissues in the TCGA cohort. **(B)** SRPX2 is significantly upregulated in tumor samples compared to paired adjacent non-tumor samples. **(C,D)** Immunohistochemical (IHC) staining of SRPX2 in adjacent normal tissues, primary tumors, and peritoneal metastatic tissues. Scatter plots indicate quantitative staining score differences. **(E)** Kaplan–Meier overall survival (OS) curves of GC patients stratified by high or low SRPX2 expression. **(F)** ROC curves predicting 1-, 3-, and 5-year prognosis based on SRPX2 expression. **(G)** ROC curve evaluating the diagnostic performance of SRPX2 expression in predicting peritoneal metastasis (PM) progression. **(H)** GSEA enrichment analysis for the high SRPX2 expression group. **(I,J)** Box plots showing GO and KEGG analyses of differentially expressed genes between high and low SRPX2 expression groups. **(K)** Box plots comparing the infiltration levels of immune and stromal cell populations between high and low SRPX2 expression groups. **(L)** Heatmap displaying correlations between SRPX2 expression and infiltration of various immune and stromal cells. **(M)** Scatter plot illustrating the correlation between SRPX2 expression and infiltration levels of immune and stromal cells in the merged GC cohort. **(N)** Scatter plots showing correlations between SRPX2 expression and CAF markers (ACTA2, FAP, PDGFRA). **(O)** UMAP clustering of single cells from a GC peritoneal metastasis sample, identifying 8 distinct cell subpopulations. **(P)** Bubble plot displaying marker gene expression for each of the 8 cell subpopulations. **(Q)** Violin/feature plot indicating SRPX2 expression localized in CAFs within the peritoneal metastasis sample. **(R)** Bubble plot highlighting high SRPX2 expression in CAFs. **(S)** Bubble plot showing elevated SRPX2 expression in single cells from peritoneal metastasis samples. **(T)** Volcano plot demonstrating significant upregulation of SRPX2 in CAFs at the single-cell level. Spearman correlation analysis was used for all correlation assessments.

Pan-cancer analysis revealed that SRPX2 is significantly overexpressed in multiple tumor types (Supplementary Figure S5A). To further investigate the role of SRPX2 in gastric cancer (GC) development and peritoneal metastasis, we analyzed its expression in GC tissues from the TCGA dataset and observed significant upregulation of SRPX2 (Figure 4A). Additional analysis of paired GC samples confirmed this finding, demonstrating markedly higher SRPX2 levels in tumor tissues compared to adjacent normal tissues (Figure 4B). We also performed immunohistochemistry (IHC) staining on clinical samples, including GC tissues with peritoneal metastasis, GC tissues without peritoneal metastasis, and adjacent normal tissues. Both staining intensity and positive staining area were significantly greater in GC tissues with or without peritoneal metastasis than in normal tissues, with the strongest intensity observed in peritoneal metastasis samples (Figures 4C,D). In the combined dataset, high SRPX2 expression was associated with poorer overall survival (OS) in GC patients (Figure 4E). ROC curve analysis indicated that SRPX2 expression had strong predictive power for long-term survival (AUC: 1-year 0.590, 3-year 0.601, 5-year 0.753) (Figure 4F). Survival analysis using the Kaplan–Meier Plotter database further confirmed that high SRPX2 expression was correlated with worse first progression (FP) and post-progression survival (PPS) (Supplementary Figures S5B,D). Using the peritoneal metastasis status of patients from the GSE15081 dataset, we constructed an ROC curve to evaluate the diagnostic value of SRPX2, which showed high accuracy in predicting peritoneal metastasis (Figure 4G). We then performed functional analysis on differentially expressed genes (DEGs) stratified by SRPX2 expression. GSEA revealed that high SRPX2 expression was associated with pathways such as ECM-receptor interaction, dilated cardiomyopathy, focal adhesion, hypertrophic cardiomyopathy (HCM), and cell adhesion molecules (CAMs) (Figure 4H). GO analysis indicated significant enrichment in molecular functions related to the extracellular matrix and extracellular structure (Figure 4I). KEGG analysis showed significant enrichment in focal adhesion, ECM-receptor interaction, and the PI3K–AKT signaling pathway (Figure 4J). These findings suggest that DEGs are primarily enriched in extracellular matrix-related pathways. To explore dynamic changes in the tumor microenvironment (TME) of GC patients with different SRPX2 expression levels, we used the MCP-counter algorithm to quantify the relative abundance of eight immune cell populations, two stromal cell populations, and epithelial cells. Box plots revealed significant differences in the infiltration levels of cytotoxic lymphocytes, T cells, B lineage cells, monocytic lineage cells, myeloid dendritic cells, endothelial cells, and fibroblasts between high- and low-SRPX2 expression groups (Figure 4K). Spearman correlation analysis showed that SRPX2 expression was significantly positively correlated with endothelial cells and cancer-associated fibroblasts (CAFs) (Figures 4L,M). Interestingly, in a peritoneal metastasis cohort, only CAFs showed a significant correlation with SRPX2 expression (Supplementary Figure S5E). Moreover, SRPX2 was significantly correlated with CAF markers including ACTA2, FAP, and PDGFRA (Figure 4N), suggesting that SRPX2 may influence CAF activity. To further investigate the distribution characteristics of SRPX2 in the TME of patients with peritoneal metastasis, we integrated single-cell RNA sequencing data from the GSE163558 and GSE183904 datasets and analyzed the relationships and gene signatures across different cell populations. Using a combination of canonical markers, we identified eight major cell clusters (Figure 4O) and further defined subclusters within them (Figure 4P). SRPX2 was widely expressed in CAFs, with higher expression levels in peritoneal metastatic GC tissues than in primary GC tissues without metastasis (Figures 4Q–S). Differential expression analysis confirmed that SRPX2 was significantly upregulated in CAFs from peritoneal metastasis patients, indicating a potential role of SRPX2 in promoting peritoneal metastasis in GC (Figure 4T). These findings preliminarily suggest that SRPX2 is highly expressed in CAFs of GC patients with peritoneal metastasis and is associated with poor prognosis, peritoneal metastasis progression, and CAF infiltration.

### SRPX2 in CAFs promotes malignant progression of GC *in vitro* and *in vivo*

**FIGURE 5:**
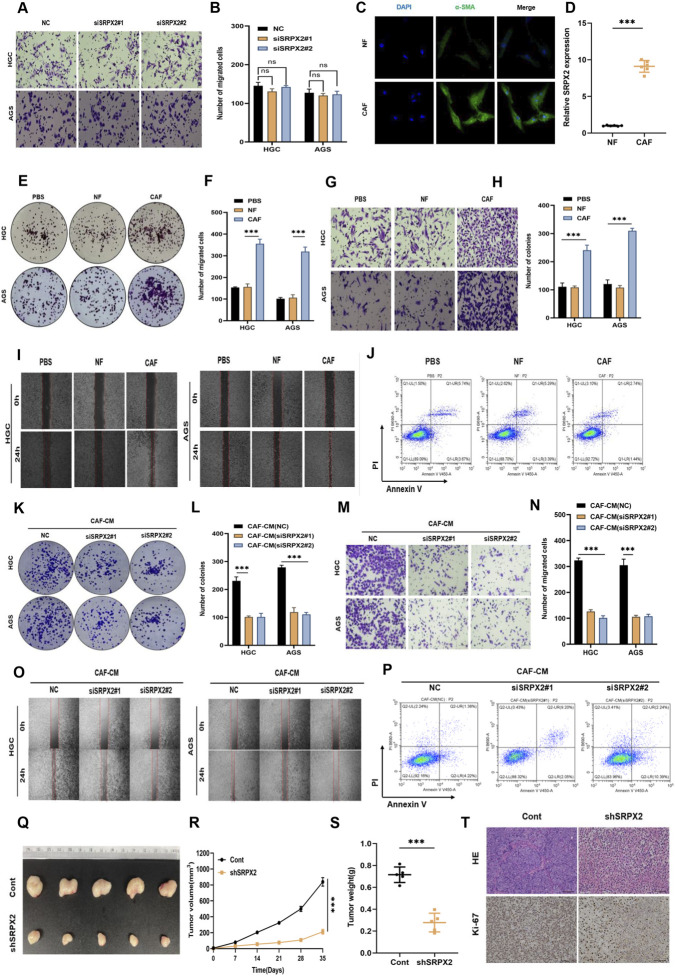
SRPX2 in CAFs promotes malignant GC progression *in vitro* and *in vivo*. **(A,B)** Transwell migration assay and quantitative analysis of HGC and AGS cells transfected with siSRPX2#1 or siSRPX2#2. **(C)** Immunofluorescence staining showing subcellular localization and expression of α-SMA in isolated CAFs and NFs. **(D)** qRT-PCR analysis of SRPX2 expression levels in isolated CAFs and NFs. **(E,F)** Colony formation assay of HGC and AGS cells co-cultured with PBS, NFs, or CAFs. **(G,H)** Transwell migration assay of HGC and AGS cells co-cultured with PBS, NFs, or CAFs. **(I)** Wound healing assay of HGC and AGS cells co-cultured with PBS, NFs, or CAFs. **(J)** Flow cytometry analysis of apoptosis in AGS cells under anoikis conditions (low-attachment culture) after co-culture with PBS, NFs, or CAFs. **(K–O)** Colony formation, Transwell migration, and wound healing assays of HGC and AGS cells cultured with conditioned medium from CAFs transfected with siSRPX2#1 or siSRPX2#2. **(P)** Flow cytometry analysis of apoptosis rates in AGS cells cultured with conditioned medium from CAFs transfected with siSRPX2#1 or siSRPX2#2. **(Q–T)** *In vivo* xenograft tumor assay: **(Q)** representative images of tumors from mice co-injected with MKN45 cells and CAFs (n = 5) or MKN45 cells and SRPX2-knockdown CAFs (shSRPX2, n = 5); **(R)** tumor growth curves; **(S)** average tumor weight; **(T)** representative H&E staining and Ki-67 immunohistochemistry of xenograft tumor sections. All data are presented as mean ± SD of triplicate experiments. ns, *P* > 0.05; ***, *P* < 0.001.

To evaluate the role of SRPX2 in GC progression and metastasis, we effectively silenced SRPX2 expression in AGS and HGC-27 cells using siRNA. Both siSRPX2#1 and siSRPX2#2 significantly suppressed SRPX2 expression. Subsequent Transwell assays were performed to assess GC cell migration. Unexpectedly, no significant difference in migration was observed between the SRPX2-knockdown and control groups (Figures 5A,B). Given our previous finding that SRPX2 is significantly correlated with CAF infiltration, we hypothesized that high SRPX2 expression in CAFs might influence GC progression. Therefore, we isolated cancer-associated fibroblasts (CAFs) from GC tissues and normal fibroblasts (NFs) from adjacent normal tissues (NATs). Immunofluorescence staining confirmed the cellular morphology and marker expression of the isolated fibroblast populations (NFs and CAFs) (Figure 5C). qRT-PCR analysis revealed that SRPX2 expression was significantly higher in CAFs than in NFs (Figure 5D). We then conducted colony formation, Transwell, and wound healing assays by co-culturing CAFs with GC cells. These experiments demonstrated that CAFs significantly enhanced the proliferation and migration abilities of GC cells (Figures 5E–I). In an *in vitro* anoikis model using low-attachment culture dishes, GC cells co-cultured with CAFs exhibited significantly lower rates of cell death compared to those co-cultured with NFs or controls, indicating enhanced anoikis resistance (Figure 5J, Supplementary Figure S5F). Therefore, we knocked down SRPX2 expression in CAFs using siSRPX2#1 or siSRPX2#2 and performed CCK-8 assays to assess their proliferative activity. The results indicated that SRPX2 knockdown did not affect the viability of CAF cells (Supplementary Figure S5G). Furthermore, when GC cells were cultured with conditioned medium from these SRPX2-knockdown CAFs, both the proliferation and migration abilities of the GC cells were significantly inhibited (Figures 5K–O). Apoptosis analysis by flow cytometry in the low-attachment model showed increased cell death in the SRPX2-knockdown group, suggesting reduced anoikis resistance (Figure 5P, Supplementary Figure S5H). To investigate the *in vivo* functional impact of SRPX2, we subcutaneously injected a mixture of CAFs and MKN45 cells (1:1 ratio) into nude mice to establish a xenograft model. Compared with the control group, knockdown of SRPX2 in CAFs effectively suppressed tumor formation and proliferation (Figure 5Q). shSRPX2 significantly inhibited both the volume and weight of subcutaneous tumors (Figures 5R,S). HE staining and Ki-67 immunohistochemical staining of the tumor tissues further confirmed this trend (Figure 5T). These findings indicate that SRPX2 in CAFs enhances the proliferation, migration, and anoikis resistance of GC cells.

### TFAP2A transcriptionally upregulates SRPX2 expression in CAFs

**FIGURE 6:**
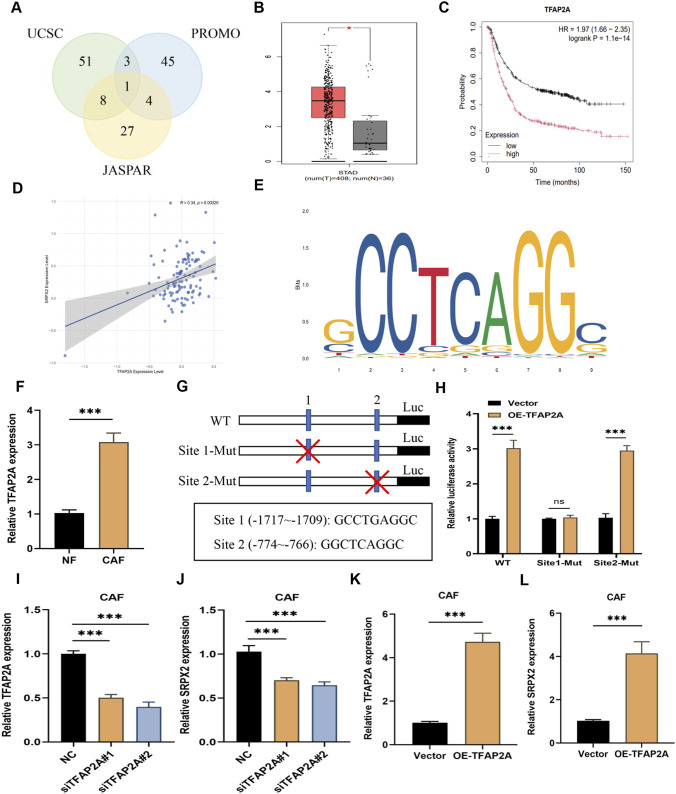
TFAP2A transcriptionally upregulates SRPX2 expression in CAFs. **(A)** Venn diagram showing the overlap of predicted transcription factors for SRPX2 using three different algorithms. **(B,C)** Analysis of TFAP2A expression in GC tumor tissues *versus* normal tissues, and Kaplan–Meier overall survival (OS) curve of GC patients stratified by high or low TFAP2A expression, using the Kaplan–Meier Plotter database. **(D)** Spearman correlation analysis of the relationship between TFAP2A and SRPX2 expression in GC tissues. **(E)** Predicted binding motif of TFAP2A to the SRPX2 promoter, as identified in the JASPAR database. **(F)** Relative expression of TFAP2A in NFs and CAFs. **(G)** Schematic of the pGL3-Basic reporter vectors containing wild-type or mutated SRPX2 promoter motifs. **(H)** Luciferase reporter assay in CAFs co-transfected with indicated mutant or wild-type pGL3-Basic vectors and control or TFAP2A expression plasmids. **(I)** Relative mRNA expression of TFAP2A in CAFs transfected with two TFAP2A-targeting siRNAs, measured by qRT-PCR. **(J)** Relative SRPX2 expression in CAFs after TFAP2A knockdown, detected by qRT-PCR. K–L. Relative mRNA expression of TFAP2A **(K)** and SRPX2 **(L)** in CAFs after TFAP2A overexpression, measured by qRT-PCR. All data are presented as mean ± SD of triplicate experiments. *, *P* < 0.05; ***, *P* < 0.001.

Given the significant upregulation of SRPX2 in CAFs, we hypothesized that SRPX2 might be regulated by an upstream transcription factor (TF) during the development of GC-associated CAFs. We retrieved the promoter sequence of SRPX2 from the UCSC Genome Browser (http://genome.ucsc.edu/) and applied three algorithms (UCSC, PROMO, and JASPAR) to predict potential TFs that may interact with this promoter region. TFAP2A was the only TF identified by all three algorithms (Figure 6A). We then examined TFAP2A expression levels in public databases and found that it is upregulated in multiple solid tumors (Supplementary Figure S5I). In gastric cancer, TFAP2A was significantly overexpressed in tumor tissues compared to normal tissues and was associated with poor prognosis (Figures 6B,C). Its expression was also positively correlated with SRPX2 levels (Figure 6D). Using the JASPAR database, we identified multiple potential TFAP2A binding sites within the SRPX2 promoter (Figure 6E). TFAP2A expression was significantly higher in CAFs than in NFs (Figure 6F). We subsequently compared the SRPX2 promoter sequence with the TFAP2A binding motif in JASPAR and predicted two possible TFAP2A binding sites within the SRPX2 promoter (Figure 6G). A dual-luciferase reporter assay was used to validate these sites. As expected, forced expression of TFAP2A significantly enhanced SRPX2 promoter activity; however, this effect was abolished when site 1—but not site 2—was mutated (Figure 6H), indicating that site 1 is required for TFAP2A-mediated regulation of SRPX2. Next, we investigated the effect of TFAP2A perturbation on SRPX2 expression. Two siRNAs targeting TFAP2A were designed for subsequent experiments (Figure 6I). Knockdown of TFAP2A significantly reduced SRPX2 expression in CAFs (Figure 6J). Conversely, overexpression of TFAP2A led to increased SRPX2 levels (Figures 6K,L). These results suggest that TFAP2A acts as a transcriptional regulator of SRPX2. In conclusion, TFAP2A may activate SRPX2 transcription in CAFs of GC patients.

### SRPX2 in CAFs activates the FAK/AKT pathway and secretes IL-6 to induce anoikis resistance in GC cells

**FIGURE 7:**
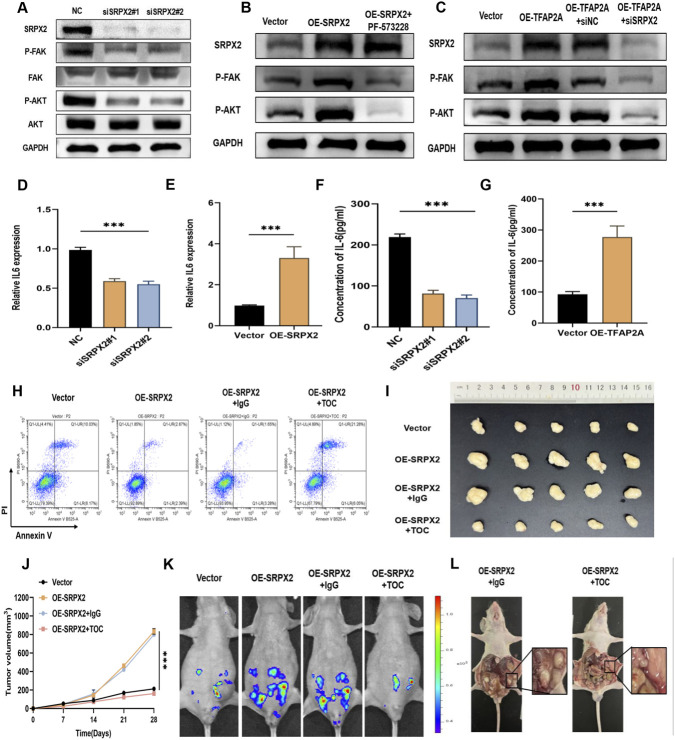
SRPX2 in CAFs activates the FAK/AKT pathway and secretes IL-6 to induce anoikis resistance in GC. **(A)** Western blot analysis of SRPX2, p-FAK, FAK, p-AKT, and AKT expression in CAFs transfected with siSRPX2#1 or siSRPX2#2. **(B)** Expression levels of p-FAK and p-AKT in CAFs overexpressing SRPX2 and treated with the FAK pathway inhibitor PF-573228 (5 μM). **(C)** Expression of SRPX2, p-FAK, and p-AKT in CAFs overexpressing TFAP2A, followed by SRPX2 knockdown. **(D,E)** Relative IL-6 mRNA expression in CAFs after SRPX2 knockdown or overexpression, measured by qRT-PCR. **(F,G)** IL-6 secretion levels in conditioned medium from CAFs after SRPX2 knockdown or overexpression, as determined by ELISA. **(H)** Apoptosis analysis by Annexin V/PI staining in AGS cells cultured under anoikis conditions (low-attachment culture) after treatment with conditioned medium from SRPX2-overexpressing CAFs pretreated with tocilizumab (10 μg/mL) for 24 h. **(I,J)** Tocilizumab treatment significantly suppressed subcutaneous tumor growth in BALB/c nude mice injected with MKN45 cells and SRPX2-overexpressing CAFs. **(K)** *In vivo* monitoring of GC cell dissemination in the abdominal cavity using IVIS imaging; tocilizumab treatment markedly inhibited peritoneal metastasis. **(L)** Representative images of peritoneal metastases in mice at the end of the study. All data are presented as mean ± SD of triplicate experiments. ***, *P* < 0.001.

To further elucidate the mechanism of SRPX2 and identify its downstream effectors, we focused on relevant signaling pathways. Previous studies have reported that SRPX2 promotes tumor cell proliferation and migration by activating FAK and AKT phosphorylation (Li et al., 2017). Consistently, our data revealed that differentially expressed genes in the SRPX2-high group were enriched in pathways such as Focal adhesion and PI3K–AKT. We therefore hypothesized that high SRPX2 expression in CAFs may regulate the activation of the FAK/AKT pathway. Western blot analysis showed that knockdown of SRPX2 in CAFs significantly reduced phosphorylated FAK and AKT levels, while total FAK and AKT protein expression remained unchanged (Figure 7A). A consistent trend was observed in subcutaneous tumor tissues from our earlier mouse model via IHC staining (Supplementary Figure S5J). In a rescue experiment, we treated SRPX2-overexpressing CAFs with the FAK pathway inhibitor PF-573228. Inhibition of the FAK pathway significantly attenuated the anoikis resistance of GC cells cultured in conditioned medium from SRPX2-overexpressing CAFs (Figure 7B, Supplementary Figure S5K,L). Furthermore, TFAP2A overexpression confirmed its role in transcriptionally activating SRPX2 and subsequently promoting FAK/AKT pathway activation (Figure 7C). To explore how CAFs influence anoikis resistance in GC cells, we considered the involvement of inflammatory cytokines. Previous studies have shown that FAK/AKT pathway activation is closely associated with IL-6 release, and that IL-6 can induce anoikis resistance in tumor cells via the JAK–STAT3 pathway (Pokharel et al., 2019; Ham et al., 2019). We therefore measured IL-6 expression and secretion in CAFs using qRT-PCR and ELISA. The results demonstrated that SRPX2 upregulation significantly enhanced IL-6 secretion from CAFs (Figures 7D–G). To determine whether IL-6 secretion directly contributes to anoikis resistance in GC cells, we used tocilizumab, an FDA-approved IL-6Rα inhibitor that blocks IL-6 binding to its membrane receptor and subsequent intracellular signaling. Flow cytometry analysis showed that tocilizumab significantly reduced anoikis resistance in GC cells (Figure 7H, Supplementary Figure S5M). We next established subcutaneous xenograft and peritoneal metastasis mouse models to evaluate the effect of tocilizumab on GC progression and metastasis. Tumors in mice co-injected with SRPX2-overexpressing CAFs exhibited significantly enhanced growth, which was reversed by tocilizumab treatment (Figures 7I,J). Similarly, inhibition of the IL-6 axis markedly reduced the formation of peritoneal metastatic nodules (Figures 7K,L). These results demonstrate that the FAK/AKT/IL-6 signaling axis can be pharmacologically targeted to suppress GC cell proliferation and peritoneal metastasis *in vivo*. Therefore, pharmacological inhibition of IL-6 represents a promising therapeutic strategy against peritoneal metastasis in GC patients by counteracting CAF-derived SRPX2-mediated anoikis resistance.

## Discussion

Anoikis, a form of programmed cell death, traditionally plays a critical role in host defense. However, the dynamic evolution of tumor malignancy often leads to the activation of anoikis resistance mechanisms, which has become a hallmark of cancer progression and metastasis (Talukdar et al., 2018). In this study, we integrated multiple public datasets to screen for anoikis-related genes (ARGs) in GC samples. Using non-negative matrix factorization (NMF) algorithm, we constructed anoikis-based molecular subtypes among GC patients. Survival analysis revealed that Subtype A was significantly associated with poor prognosis and highly expressed established promoters of anoikis resistance, such as MMP11, TIMP1, SERPINE1, NOTCH3, ANGPTL4, and PDGFRB (Takeuchi et al., 2011; Toricelli et al., 2013; Polo-Generelo et al., 2024; Brown et al., 2015; Shen et al., 2017). We therefore concluded that anoikis resistance is markedly activated in Subtype A, contributing to unfavorable patient outcomes. We further applied WGCNA to identify 169 potential markers closely associated with anoikis resistance in Subtype A. Based on these, we developed and internally/externally validated a robust anoikis resistance risk model comprising six key genes, underscoring the importance of ARGs in diagnosing and prognosticating GC. Given that anoikis resistance supports the survival and dissemination of suspended cells and maintains highly invasive biological properties through morphological adaptations (Du et al., 2018), and considering the critical role of free tumor cell survival in the peritoneal cavity in the pathogenesis of gastric peritoneal metastasis (PM) (Mikuła-Pietrasik et al., 2018), we innovatively constructed a PM risk diagnostic model using the random forest (RF) algorithm. This model demonstrated high specificity and sensitivity, offering a valuable clinical decision-making tool for diagnosing PM progression in GC patients.

Our prognostic risk model incorporated six key factors—HEYL, SRPX2, LBH, PLAT, ITGAV, and HTRA1—all implicated in tumor progression. For instance, HEYL, a direct target of the Notch pathway, promotes breast cancer proliferation by suppressing TGF-β signaling via interaction with Smad proteins (Han et al., 2014). In colorectal cancer, SRPX2 protein stability is regulated by circSEC24B, inducing chemotherapy resistance (Wang et al., 2024). Exosomal LBH in nasopharyngeal carcinoma modulates VEGFA to suppress EMT and angiogenesis in the tumor microenvironment (Wu et al., 2022). PLAT is significantly associated with prognosis in glioblastoma and low-grade glioma (Zhang et al., 2024). ITGAV activates TGF-β and drives EMT in pancreatic cancer; its specific inhibition curbs peritoneal carcinomatosis, tumor growth, and distant metastasis (Kemper et al., 2021). Downregulation of HTRA1 in ovarian cancer alleviates EGFR suppression-induced anoikis resistance and ultimately promotes intraperitoneal dissemination (He et al., 2010). Similarly, each factor in our PM diagnostic model has been linked to malignant progression and metastasis. ZFHX4 is associated with metastasis and poor prognosis in osteosarcoma (Pires et al., 2023). Mutations in the CYTB locus influence adrenal metastasis in renal cell carcinoma (Komiyama et al., 2022). Dai et al. reported that downregulation of CLEC3B in hepatocellular carcinoma mediates AMPK and VEGF signaling, promoting migration, invasion, and EMT in tumor and endothelial cells (Dai et al., 2019), consistent with the negative risk association of CLEC3B in our model. CHRDL2 has been shown to activate YAP/TAZ signaling to facilitate gastric cancer metastasis (Che et al., 2024). However, the mechanistic role of SRPX2 in gastric cancer peritoneal metastasis remained unclear.

SRPX2 was the only key gene identified in both the GC prognostic model and the PM diagnostic model. Analyses of public datasets revealed SRPX2 upregulation in tumor tissues compared to adjacent normal samples, which we validated via IHC staining of clinical GC specimens. Furthermore, high SRPX2 expression correlated with poor prognosis, and ROC analysis indicated its strong predictive value for both overall survival and PM progression. Interestingly, *in vivo* experiments showed that SRPX2 did not affect GC cell migration. Instead, comprehensive analysis of stromal and immune cells in the tumor microenvironment (TME) revealed that SRPX2 expression was significantly correlated with CAF marker expression and infiltration levels in both primary and PM GC samples. Integrated single-cell data further confirmed prominent SRPX2 expression in CAFs from PM samples.Previous studies have shown that CAFs in breast cancer promote anoikis resistance in ECM-detached cells via secretion of IGFBP (Weigel et al., 2014). Similarly, we found that SRPX2 was highly expressed in CAFs isolated from GC tissues, and co-culture with these CAFs significantly enhanced GC cell proliferation, migration, and apoptosis resistance. Given that upregulation of oncogenic factors in tumor cells is often regulated epigenetically, we identified TFAP2A as a transcription factor promoting SRPX2 transcription in CAFs. TFAP2A has been reported to exert oncogenic effects in other cancers (Berl et al., 2011; Shi et al., 2014), and we confirmed its association with poor prognosis in GC.

To elucidate the functional mechanism of SRPX2 in CAFs, we performed functional enrichment analysis, which revealed significant involvement of focal adhesion, PI3K/AKT signaling, and ECM-receptor interaction pathways. Previous studies indicate that Talin1, a focal adhesion complex protein, regulates anoikis resistance in detached cells by phosphorylating adhesion molecules and activating downstream AKT, enhancing adhesion, migration, and invasion in prostate cancer (Sakamoto et al., 2010). SRPX2 mediates MMP2/9 secretion via the FAK/AKT pathway to promote migration and invasion in hepatocellular carcinoma (Li et al., 2017). Activation of the FAK pathway in epithelial cells is also associated with release of pro-inflammatory cytokines such as TNF and IL-6 (Pokharel et al., 2019). We confirmed that knockdown of SRPX2 in CAFs downregulated FAK/AKT signaling. Moreover, inhibition of the FAK pathway in SRPX2-overexpressing CAFs mitigated their pro-anoikis effects on GC cells. Interestingly, we also observed significantly elevated IL-6 transcription and secretion in the conditioned medium of high-SRPX2 CAFs, which was reversed upon SRPX2 knockdown. Treatment with tocilizumab, an IL-6Rα inhibitor, reduced anoikis resistance in GC cells *in vitro* and suppressed tumor growth and peritoneal metastasis *in vivo*, demonstrating the functional role of IL-6 secretion. These results indicate that SRPX2-high CAFs in the GC microenvironment activate the FAK/AKT pathway to promote IL-6 paracrine signaling, inducing anoikis resistance in GC cells. Tocilizumab may represent a promising clinical agent for delaying GC progression and peritoneal metastasis.

## Conclusion

In summary, we have identified a novel anoikis-related molecular subtype in gastric cancer and developed robust prognostic and peritoneal metastasis (PM) diagnostic models. We further demonstrated that high expression of SRPX2 in cancer-associated fibroblasts (CAFs) within the tumor microenvironment promotes IL-6 secretion via activation of the FAK/AKT pathway, thereby inducing anoikis resistance in GC cells and facilitating peritoneal metastasis. Inhibition of the IL-6 signaling axis using tocilizumab effectively suppressed GC progression and peritoneal dissemination. These findings suggest that targeting the interaction between cancer cells and CAFs through tocilizumab may hold clinical potential for the treatment of gastric cancer.

## Data Availability

The datasets generated and analyzed during this study are derived from the following publicly available repositories: The Cancer Genome Atlas (TCGA) database (https://portal.gdc.cancer.gov/) and the Gene Expression Omnibus (GEO) (https://www.ncbi.nlm.nih.gov/geo/) under accession numbers GSE65801, GSE84426, GSE84433, GSE38749, GSE15081, GSE163558, and GSE183904. The list of potential anoikis resistance genes was sourced from GeneCards (https://www.genecards.org/). Additional supporting data and results from this study are available from the corresponding author upon reasonable request.

## Supplementary material

SUPPLEMENTARY FIGURE S1
**(A)** Heatmap showing differential expression of 87 ARGs between tumor and normal samples in the TCGA-STAD cohort. **(B)** Volcano plot of differentially expressed ARGs. **(C)** Heatmap of differentially expressed genes in paired normal and GC tissues from the GSE65801 dataset. **(D)** Venn diagram of overlapping differentially expressed ARGs between the TCGA-STAD cohort and the GSE65801 dataset. **(E)** Mutation frequency of differentially expressed ARGs in GC samples from the TCGA-STAD cohort. **(F)** Copy number variation (CNV) profile of 42 differentially expressed ARGs. **(G)** Chromosomal locations of CNV alterations in the 42 differential ARGs.

SUPPLEMENTARY FIGURE S2
**(A)** Protein-protein interaction network among differentially expressed ARGs. Circle color indicates prognostic risk type; circle size reflects the strength of prognostic association. **(B)** Forest plot showing prognostic correlations of differential ARGs. **(C)** Consensus clustering matrix from NMF analysis of GC samples based on ARG expression. **(D)** Selection of optimal cluster number using cophenetic and dispersion coefficients. **(E)** Clustering subpopulation distribution diagrams based on PCA, t-SNE, and UMAP analysis of 14 anoikis-related gene expression profiles. **(F)** Overall survival (OS) curves for the two subtypes. **(G)** Differential expression of prognosis-associated ARGs between the two subtypes. **(H)** Heatmap of prognostic ARG expression combined with clinicopathological features in the two subtypes. **(I)** Differences in immune cell infiltration abundance between the two subtypes. **(J)** GSVA enrichment analysis of signaling pathways in the two subtypes. **(K)** Gene set enrichment analysis (GSEA) for subtype A. *, *P* < 0.05, **, *P* < 0.01, ***, *P* < 0.001.

SUPPLEMENTARY FIGURE S3
**(A)** Sample clustering dendrogram with outlier removal. **(B)** Network topology analysis for soft-thresholding power selection. **(C)** Gene clustering dendrogram with color-assigned modules. **(D)** Heatmap of module–trait relationships between identified gene modules and subtypes A and B. **(E)**. Scatter plot of module membership (MM) versus gene significance (GS) for the blue module in subtype A.

SUPPLEMENTARY FIGURE S4
**(A)** Heatmap of risk factor expression, risk score distribution, and patient survival status scatter plots in high and low risk groups in the internal validation set. **(B,C)** Box plots comparing risk scores across different Age and Gender.

SUPPLEMENTARY FIGURE S5
**(A)** Expression of SRPX2 across various cancer types based on the Kaplan‐Meier Plotter database. **(B–D)** Kaplan–Meier survival curves for gastric cancer (GC) patients stratified by high or low SRPX2 expression, showing overall survival (OS), first progression (FP), and post-progression survival (PPS) from the Kaplan-Meier Plotter database. **(E)** Scatter plots showing correlations between SRPX2 expression and infiltration levels of immune and stromal cells in the GSE15081 dataset; Spearman correlation analysis was used. **(F)** Quantitative analysis of apoptotic cells (%) in AGS cells under anoikis conditions (low-attachment culture) after co-culture with PBS, NFs, or CAFs, as determined by flow cytometry. **(G)** The proliferative ability of CAFs was determined using the CCK-8 assay. **(H)** Quantitative analysis of apoptotic cells (%) in AGS cells cultured with conditioned medium from CAFs transfected with siSRPX2#1 or siSRPX2#2, as determined by flow cytometry. **(I)** Expression of TFAP2A across various cancer types based on the Kaplan-Meier Plotter database. **(J)** Immunohistochemical staining of SRPX2, p-FAK, and p-AKT in xenograft tumor tissues from mice co-injected with MKN45 cells and CAFs (n=5) or MKN45 cells and SRPX2-knockdown CAFs (n=5). (K,L) Flow cytometry analysis and quantitative analysis of apoptotic cells (%) in AGS cells under anoikis conditions after culture with conditioned medium from SRPX2-overexpressing CAFs treated with the FAK pathway inhibitor PF-573228 (5 μM). (M) Histogram of apoptosis analysis by Annexin V/PI staining in AGS cells under anoikis conditions (low-attachment culture) after culture with conditioned medium from SRPX2-overexpressing CAFs pretreated with tocilizumab (10 μg/ml) for 24 hours. All data are presented as the mean ± SD of triplicate experiments. *, *P* < 0.05; **, *P* < 0.01; ***, *P* < 0.001.
