# Supplementary figures and images for: Systematic analysis of anoikis-related genes identifies SRPX2-FAK/AKT-IL-6 axis in the progression and peritoneal metastasis of gastric cancer
Source: Front Genet. 2026 Jan 8;16:1736097. doi: 10.3389/fgene.2025.1736097 (PMC12835628; doi:10.3389/fgene.2025.1736097)

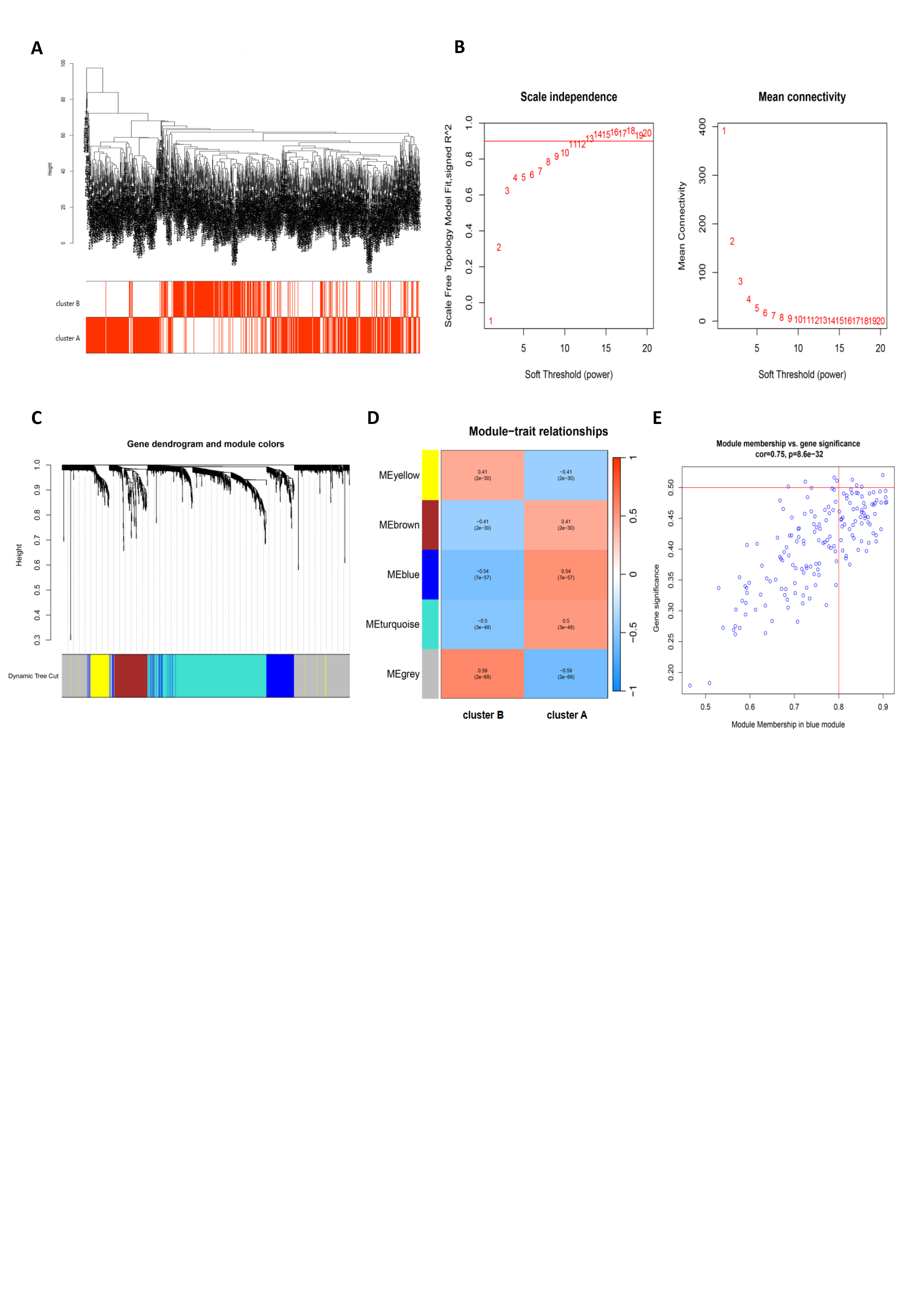

Supplement: Supplementary file 1 [file Image3.tif]

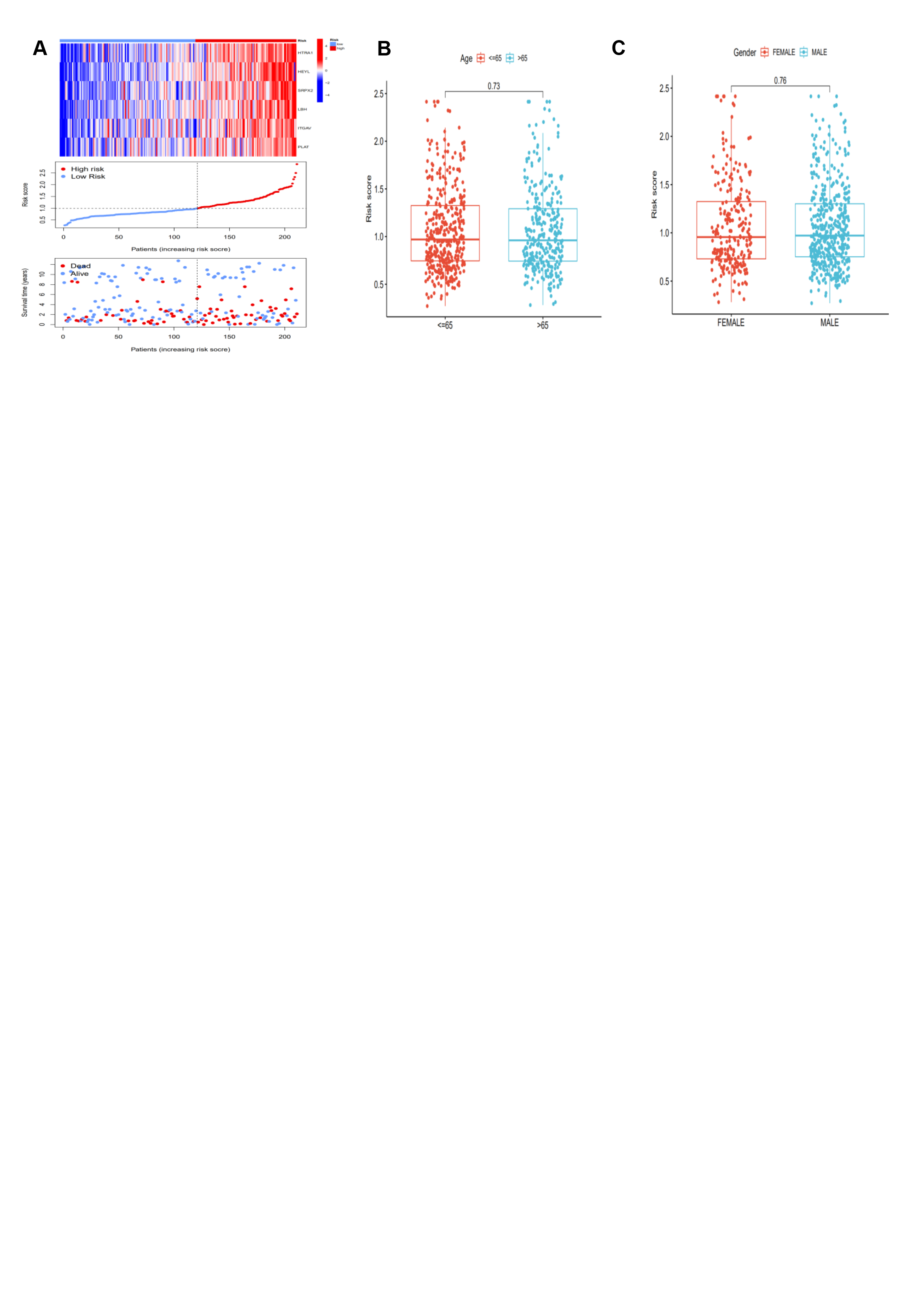

Supplement: Supplementary file 2 [file Image4.tif]

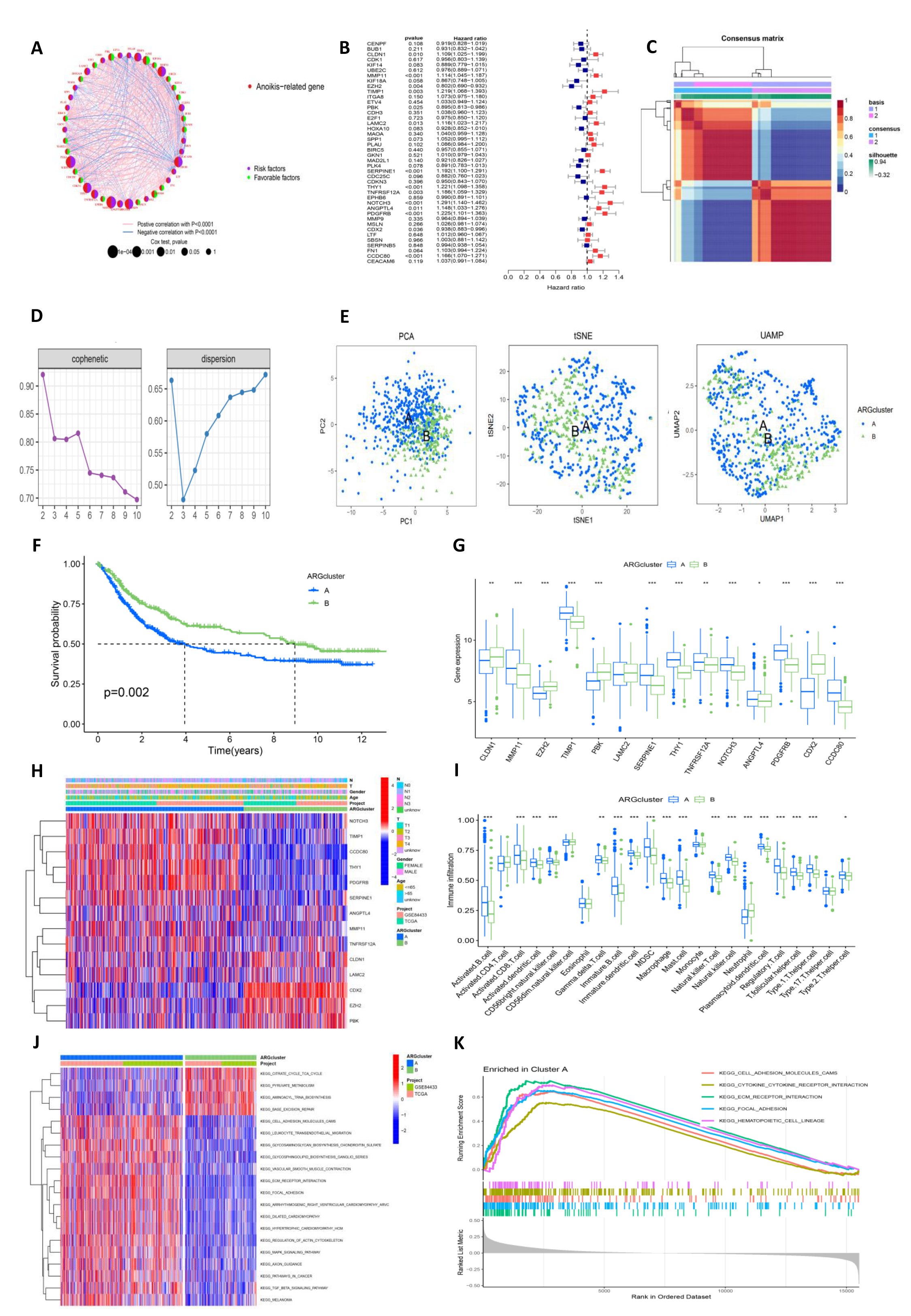

Supplement: Supplementary file 3 [file Image2.tif]

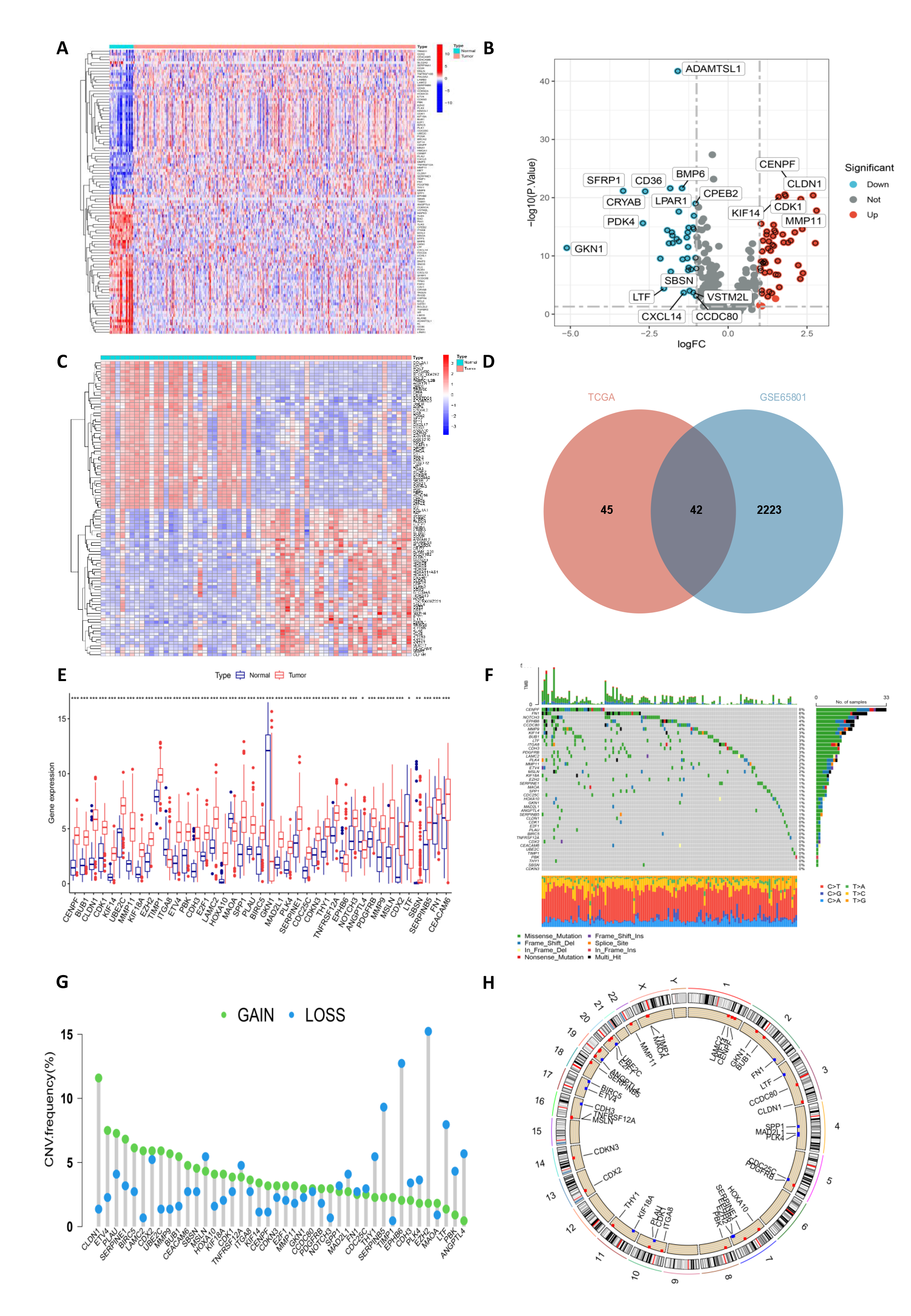

Supplement: Supplementary file 4 [file Image1.tif]

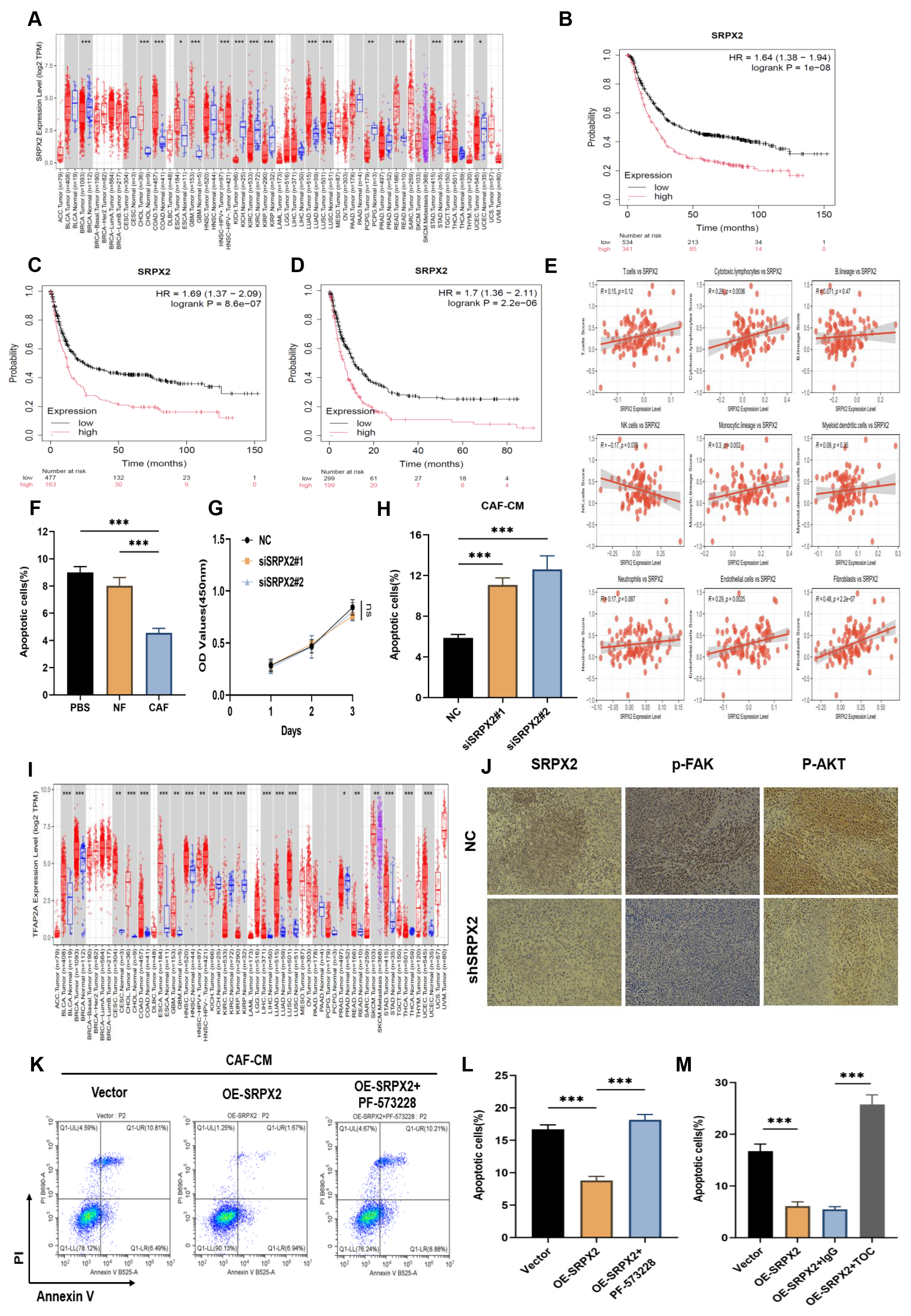

Supplement: Supplementary file 6 [file Image5.tif]
