# Supplementary material for: Systematic analysis of anoikis-related genes identifies SRPX2-FAK/AKT-IL-6 axis in the progression and peritoneal metastasis of gastric cancer
Source: Front Genet. 2026 Jan 8;16:1736097. doi: 10.3389/fgene.2025.1736097 (PMC12835628; doi:10.3389/fgene.2025.1736097)

Figure 7A

Normal

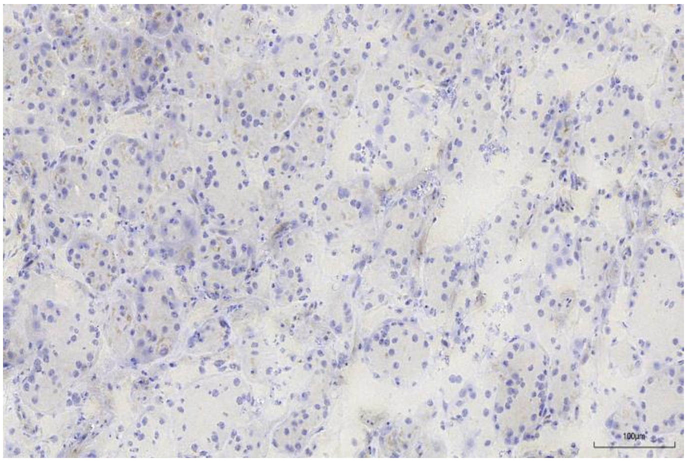

Tumor

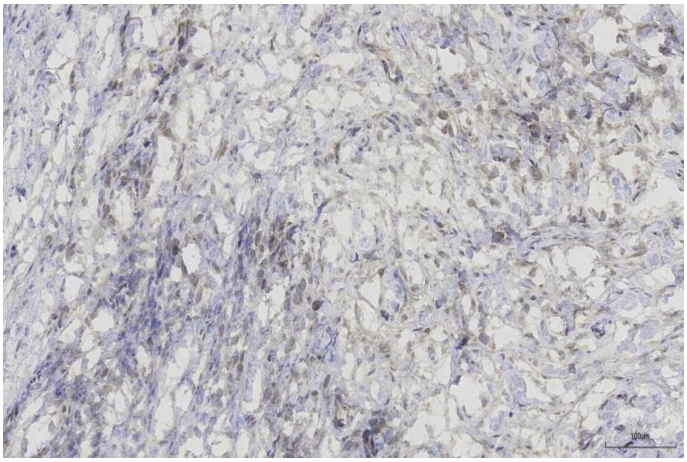

PM

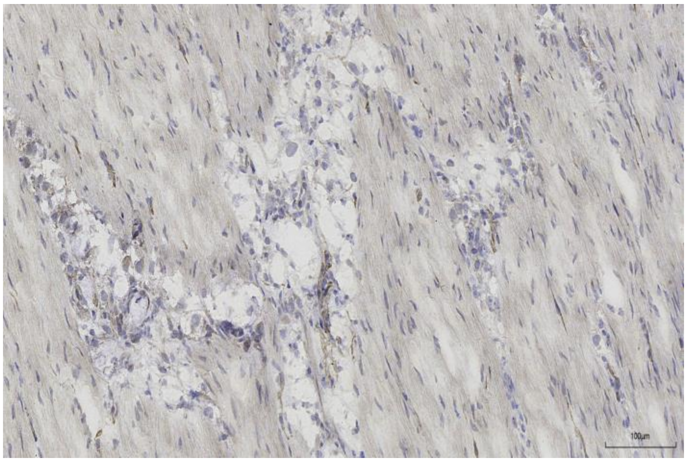

Figure8E

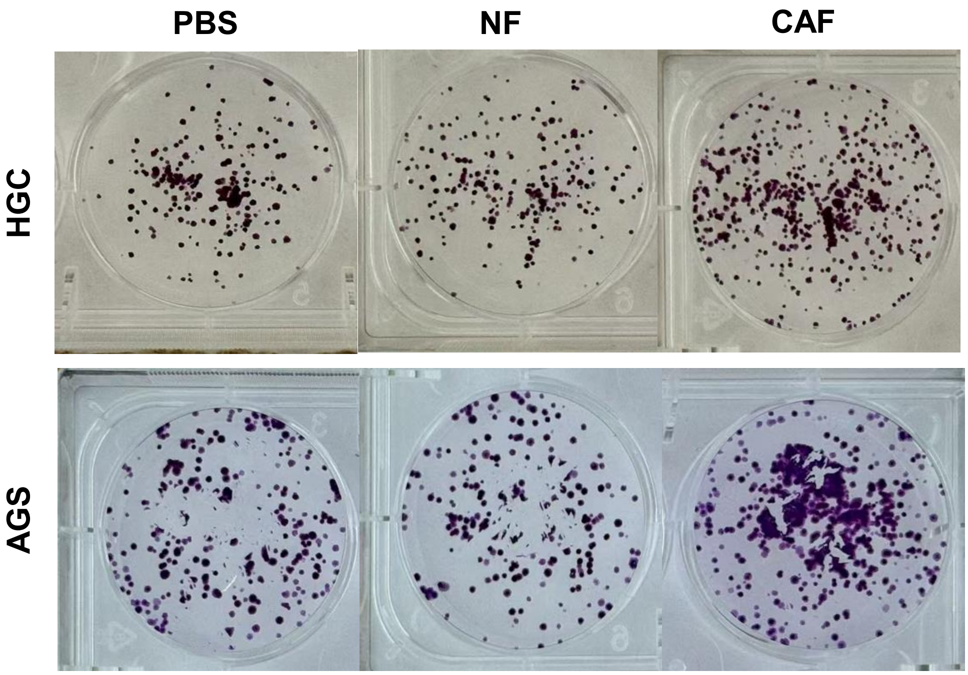

Figure8K

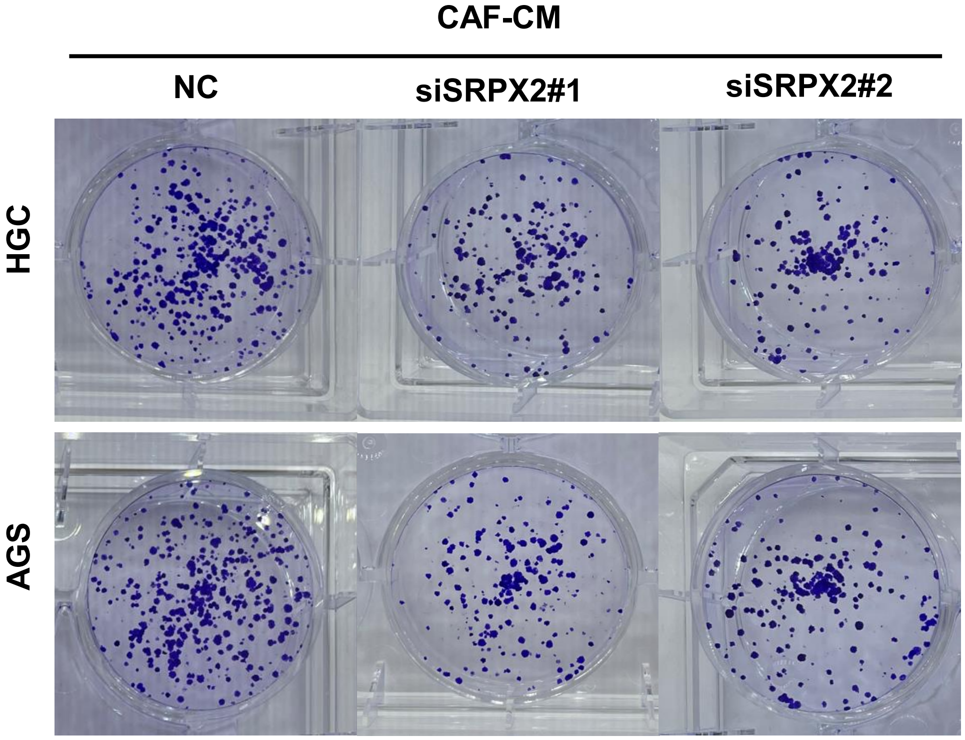

Figure8T

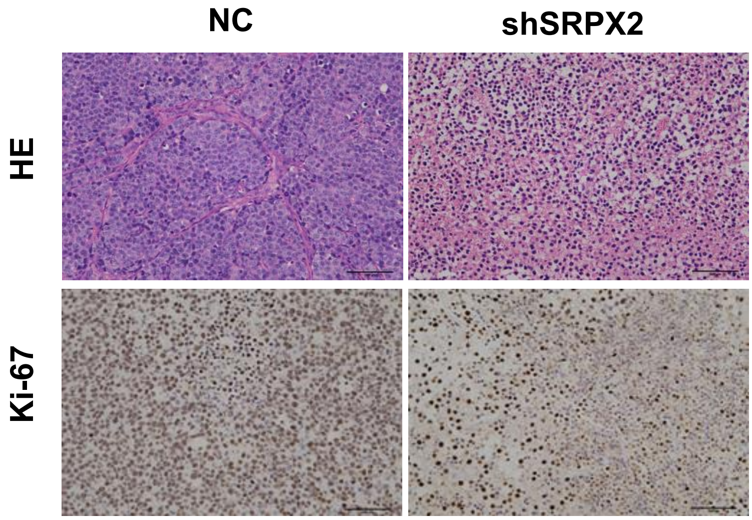

Figure10A 180 kDa Prestained Protein Marker (Vazyme Biotech Co., Ltd., product no. MP102)

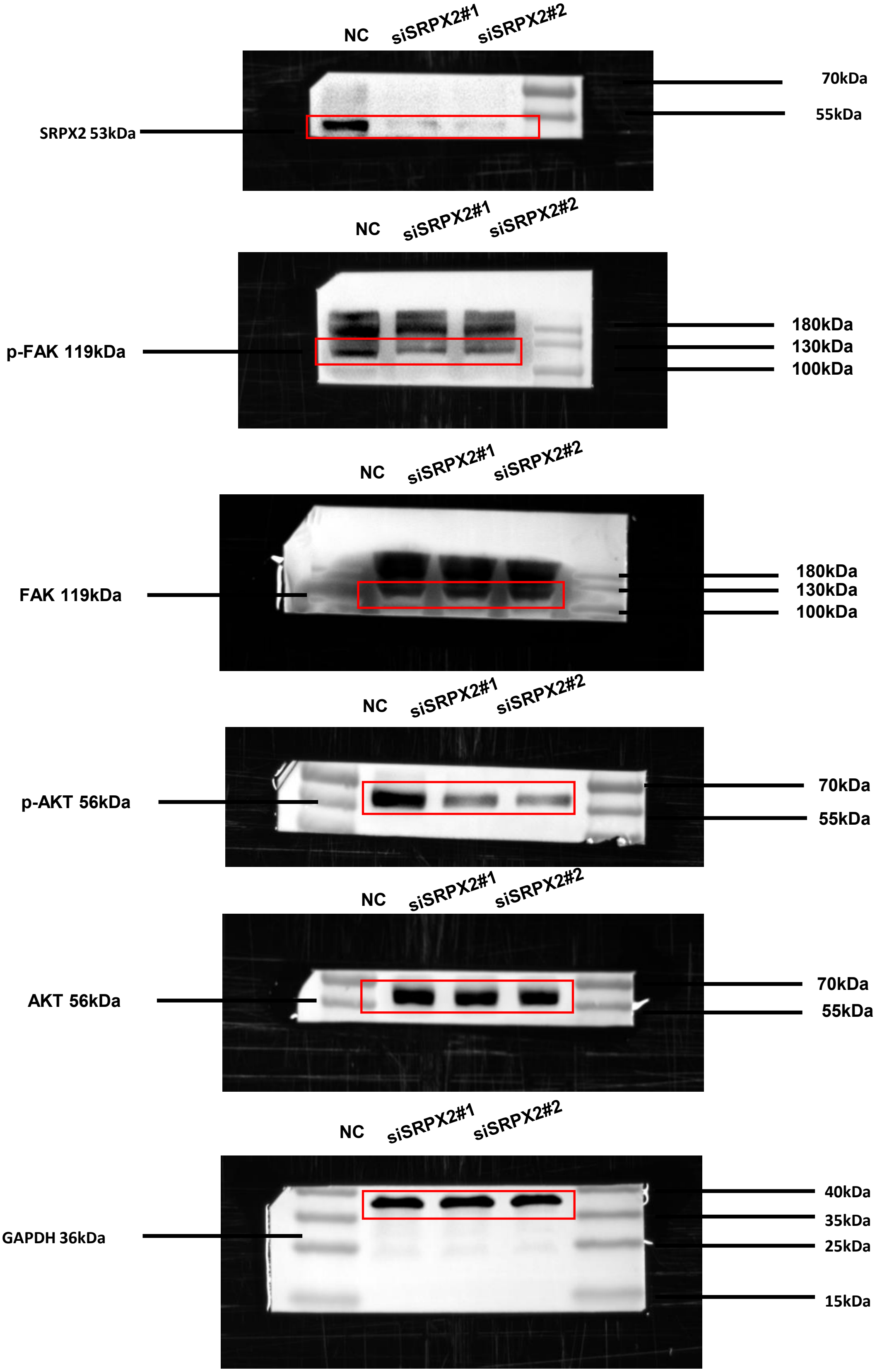

Figure10B 180 kDa Prestained Protein Marker (Vazyme Biotech Co., Ltd., product no. MP102)

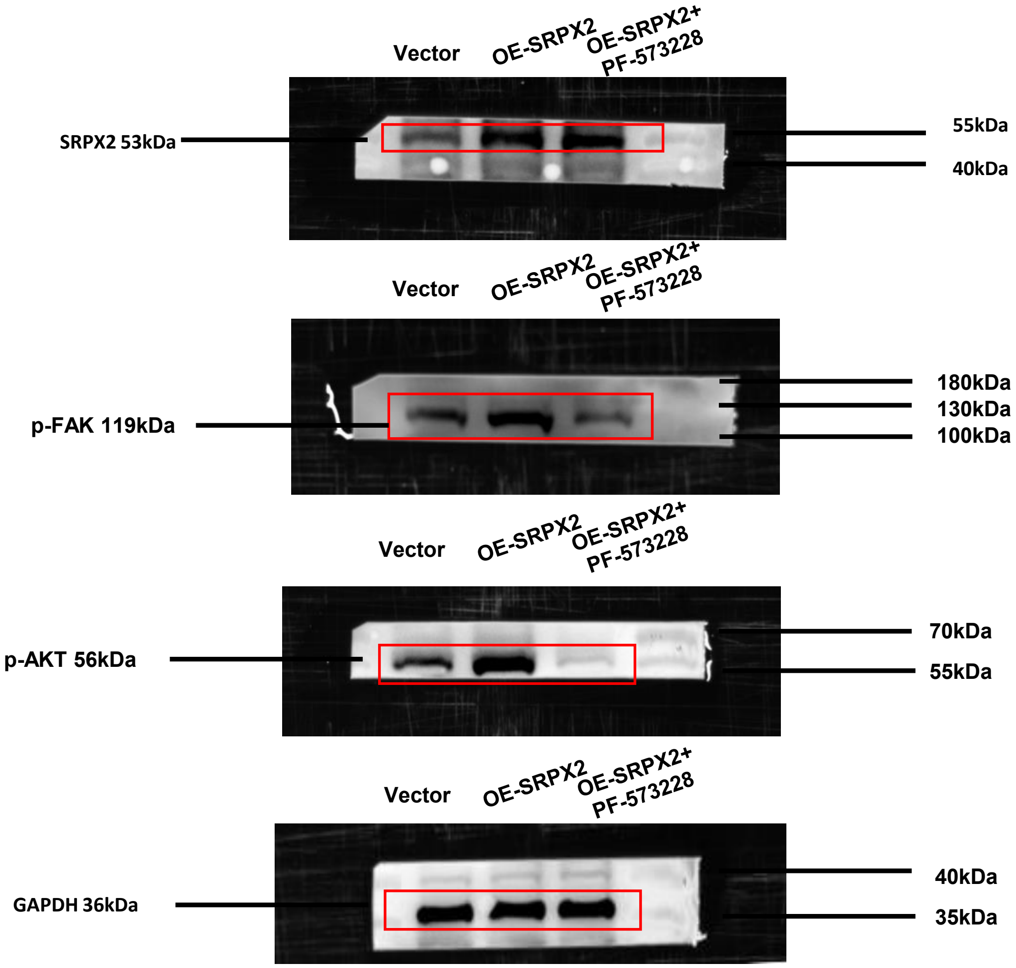

Figure10C 180 kDa Prestained Protein Marker (Vazyme Biotech Co., Ltd., product no. MP102)

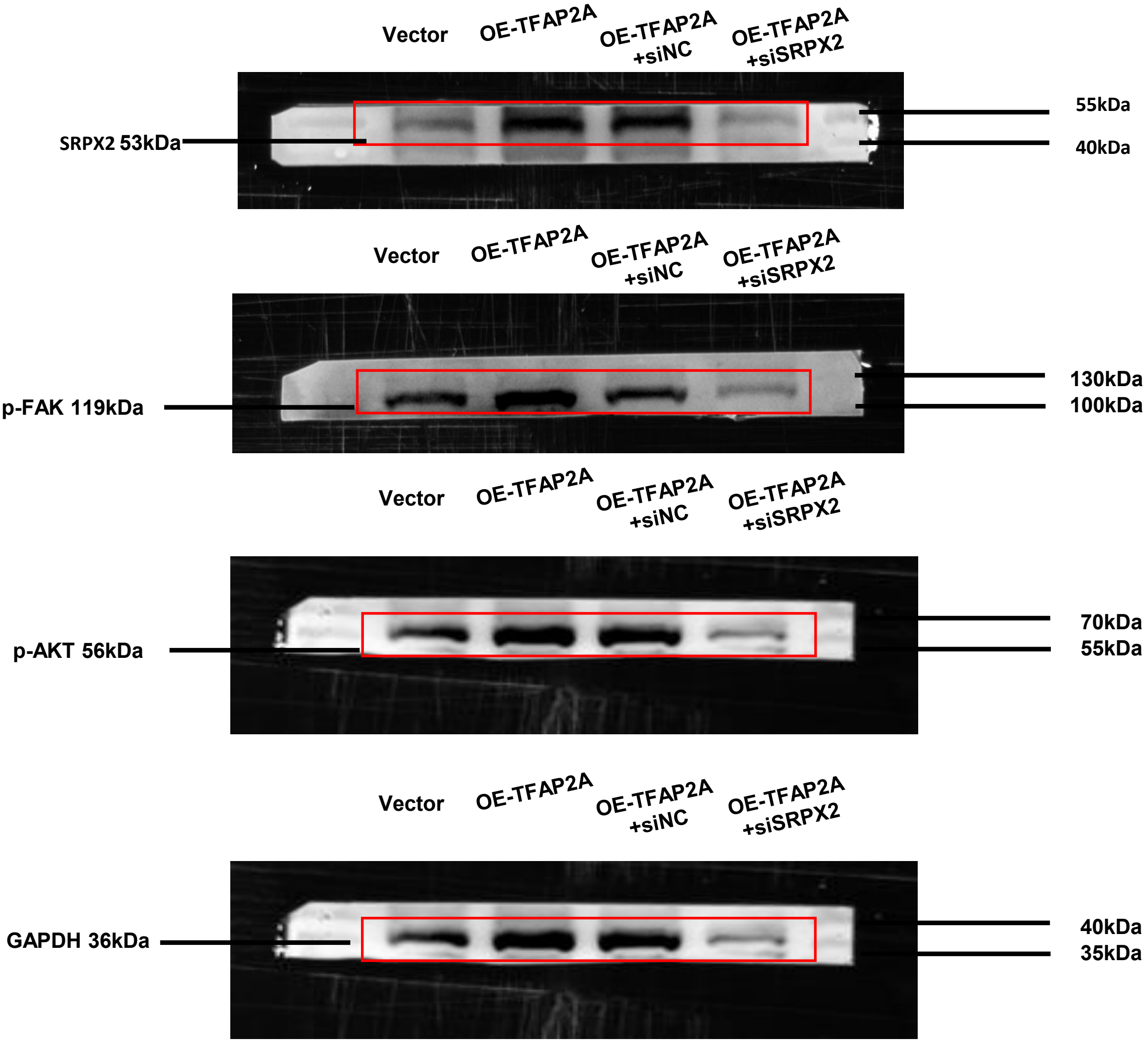

Supplementary Figure S2 H

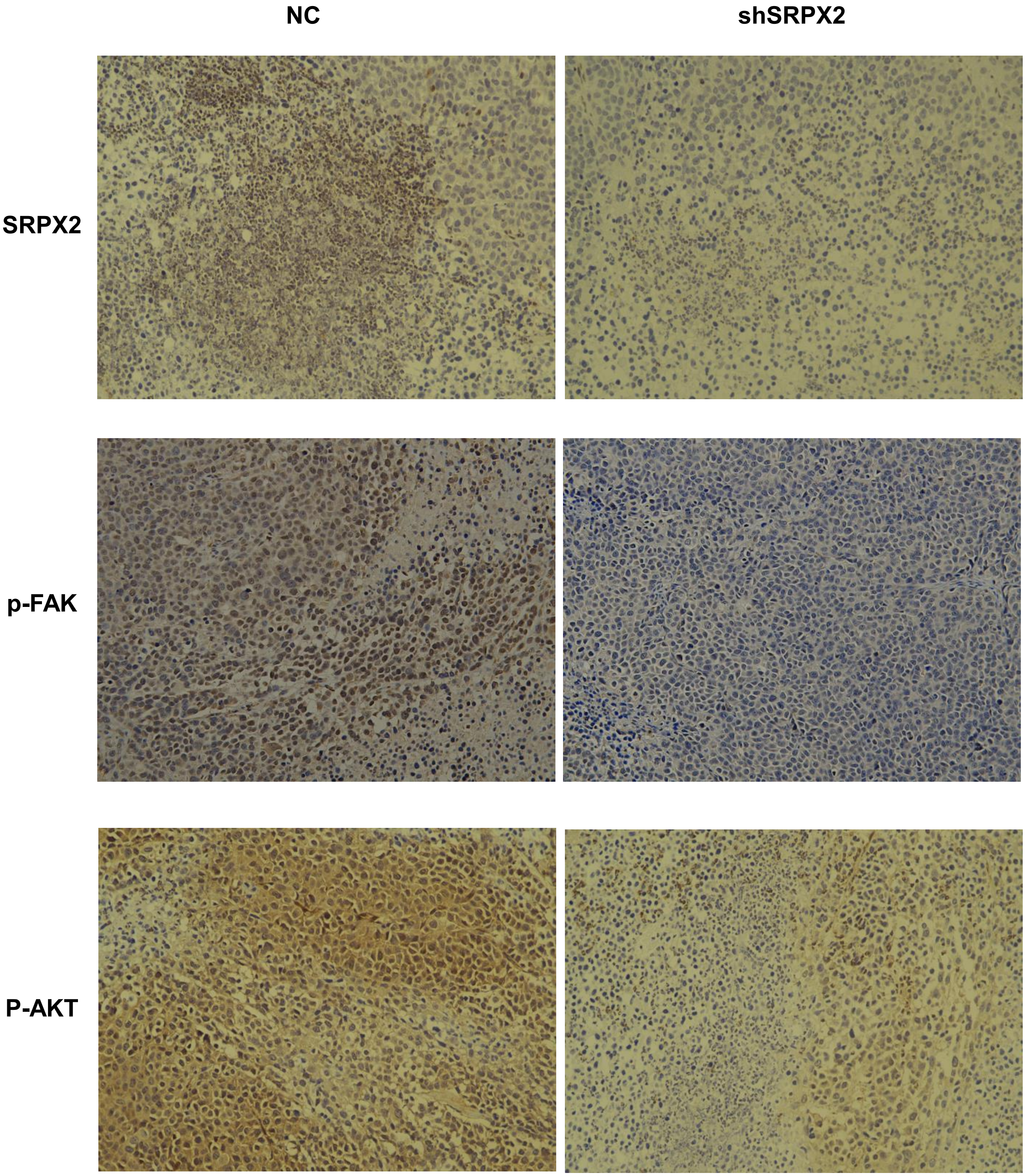

Supplement: Supplementary file 5 [file DataSheet1.pdf]
